# Rat liver folate metabolism can provide an independent functioning of associated metabolic pathways

**DOI:** 10.1038/s41598-019-44009-5

**Published:** 2019-05-21

**Authors:** Aleksandr V. Zaitsev, Michael V. Martinov, Victor M. Vitvitsky, Fazoil I. Ataullakhanov

**Affiliations:** 1 Department of Physics, Moscow State University, Moscow, 119991 Russia; 2 Center for Theoretical Problems of Physico-Chemical Pharmacology, Russian Academy of Sciences, Moscow, 119991 Russia; 3 Dmitry Rogachev National Medical Research Center for Pediatric Hematology, Oncology, and Immunology, Moscow, 117997 Russia

**Keywords:** Metabolic pathways, Networks and systems biology, Biochemical reaction networks, Computational models

## Abstract

Folate metabolism in mammalian cells is essential for multiple vital processes, including purine and pyrimidine synthesis, histidine catabolism, methionine recycling, and utilization of formic acid. It remains unknown, however, whether these processes affect each other via folate metabolism or can function independently based on cellular needs. We addressed this question using a quantitative mathematical model of folate metabolism in rat liver cytoplasm. Variation in the rates of metabolic processes associated with folate metabolism (i.e., purine and pyrimidine synthesis, histidine catabolism, and influxes of formate and methionine) in the model revealed that folate metabolism is organized in a striking manner that enables activation or inhibition of each individual process independently of the metabolic fluxes in others. In mechanistic terms, this independence is based on the high activities of a group of enzymes involved in folate metabolism, which efficiently maintain close-to-equilibrium ratios between substrates and products of enzymatic reactions.

## Introduction

**Figure 1:**
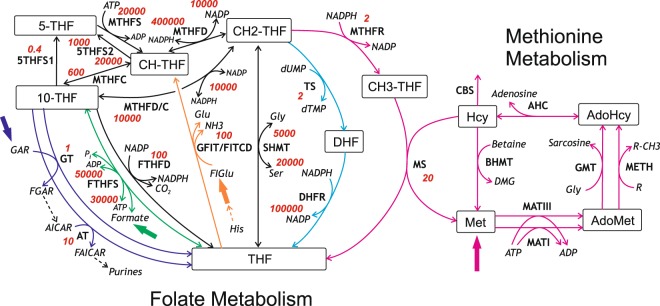
Folate and methionine metabolism in cytoplasm of rat hepatocytes. Thin solid arrows indicate reactions. Dashed arrows indicate reaction sequences. Bold arrows indicate influxes of non-folate input metabolites for the main metabolic processes associated with folate metabolism. Violet, green, orange cyan, and magenta arrows indicate reactions or metabolic fluxes associated with purine synthesis, formate utilization, histidine catabolism, pyrimidine synthesis, and methionine metabolism, respectively. Names of folates and intermediates of methionine metabolism are shown in square boxes. Other metabolite names are shown in italic font. Abbreviations of enzyme/reaction names are shown in regular font. All abbreviations are listed in Table 1. Red numbers adjacent to enzyme/reaction names show activity coefficients for the corresponding enzymes/reactions. The activity coefficient was calculated for each reaction as the derivative of the reaction rate with respect to concentration of the corresponding folate substrate at physiological concentrations of all metabolites. For reversible reactions, activity coefficients are shown for the forward and reverse reactions. These coefficients characterize how the rates of reactions respond to changes in the concentrations of the corresponding folates.

Folate metabolism is mediated by a network of enzymatic reactions that transfer one-carbon groups in multiple biochemical processes. In these reactions, folates, which are various forms of folic acid (pteroyl glutamate), function as donors and acceptors of one-carbon groups. In mammalian cells, folate metabolism is involved in purine and pyrimidine synthesis, utilization of formic acid, histidine catabolism, and recycling of methionine from homocysteine^1^ (Fig. 1), and also has important implications for human health. Disturbances in folate metabolism are associated with a number of pathologies in humans, such as anemia, neural tube defects, cardiovascular diseases, and cancer^2–8^. Moreover, several components of folate metabolism have been targeted by antibiotics or anticancer drugs^9–12^. Thus, investigation of folate metabolism regulation is of great importance for improving our understanding of the mechanisms of numerous pathologies, as well as for rational development of new drugs. However, despite its great significance, the regulatory mechanisms of folate metabolism are not fully understood.

Mammalians cannot synthetize folates *de novo*, and therefore must obtain them from food and intestinal bacterial flora. To become metabolically active, folates must be covalently bound to glutamate. The main form of folate circulating in the blood is the monoglutamate of 5-methyltetrahydrofolate, which is actively transported into cells by specific transporters. In the cytoplasm, folates are converted to polyglutamate forms that contain five or six glutamate residues per folate molecule, preventing their transport from the cell and significantly increasing their affinity for folate-related enzymes^13^. Under physiological conditions, folates undergo slow, non-enzymatic oxidation and degradation, which probably governs the rate of folate efflux from cells. The sum of intracellular folate concentrations forms the intracellular folate pool available for multiple intracellular metabolic processes. The rate of each metabolic process associated with folate metabolism is determined by cellular needs for the corresponding metabolites. It remains unknown, however, whether the associated biochemical processes can function independently or are coupled to each other via folate metabolism. In this study, we addressed this question using a quantitative mathematical model of folate metabolism.

Mathematical modeling is a powerful tool for analysis of complex metabolic systems^14–17^. Several previously developed mathematical models of folate metabolism were aimed at understanding the mechanism of methotrexate action in cancer cells and optimization of antitumor therapy^18–24^ or describing folate metabolism in microorganisms with the goal of developing new antifolate drugs^25^. The most extensive modeling of folate metabolism and its connection with other metabolic systems in animal cells was performed in a series of studies published by Nijhout, Reed, and coauthors^26–31^. A more recent approach to analysis of folate metabolism utilized a hybrid stochastic model to more accurately describe the metabolic system at very low folate concentrations^32^.

The models mentioned above can simulate various aspects of folate metabolism. However, in order to study the interdependence of different metabolic processes associated with folate metabolism, we needed to (i) develop a new model that incorporates fluxes of all associated metabolic processes as input parameters, and (ii) utilize a consistent set of species and tissue-specific parameters that would enable rigorous quantitative analysis. Accordingly, for this study, we developed a quantitative mathematical model of cytosolic folate metabolism in rat liver that includes all reactions of folate and methionine metabolism in hepatocytes as well as the first reaction of the transsulfuration pathway, which is catalyzed by cystathionine β-synthase. Most of the model parameters, including all enzyme activities, were based on published data for rat liver or hepatocytes. The rates of purine and dTMP synthesis, histidine degradation, and influxes of formate and methionine (Fig. 1) were used as the model’s input parameters.

Using this model, we studied the dependence of steady-state metabolite concentrations and reaction rates on the size of the folate pool and the effects of activation of each metabolic process associated with folate metabolism on the other processes and folate metabolism in general. Analysis of the model revealed that a group of highly active enzymes involved in folate metabolism, SHMT, MTHFC, and MTHFD, maintain the corresponding reversible reactions near equilibrium, providing the opportunity to activate or inhibit each individual metabolic process while exerting a minimal impact on the other processes in the system. Additionally, the model made several unexpected predictions regarding folate metabolism. First, liver cytoplasmic folate metabolism can maintain physiological formate levels even in the absence of an external source of formate. Second, folate metabolism ensures that vital metabolic processes continue at their physiological rates even when the size of the folate pool drops significantly below physiological levels. Third, although deficiency in folates causes an increase in homocysteine levels (a risk factor for cardiovascular diseases)^33–37^, the model predicted that this increase is significantly smaller in the liver than in tissues lacking the transsulfuration pathway, such as heart muscle.

## Methods

### Model description

The model consists of two parts, which describe metabolism of folates and methionine. The part of the model describing the kinetics of the concentrations of folates and associated metabolites is a system of ordinary differential equations constructed according to the scheme shown in Fig. 1:

1 $$\begin{array}{c}\frac{d[THF]}{dt}={V}^{DHFR}+{V}^{AT}+{V}^{GT}+{V}^{MS}+{V}^{FTHFD}-{V}^{SHMT}-{V}^{GFIT/FITCD}-{V}^{FTHFS}\\ \frac{d[CH2-THF]}{dt}=-\,{V}^{MTHFD/C}-{V}^{MTHFD}+{V}^{SHMT}-{V}^{MTHFR}-{V}^{TS}\\ \frac{d[10-THF]}{dt}={V}^{MTHFD/C}+{V}^{MTHFC}+{V}^{FTHFS}-{V}^{5THFS1}-{V}^{FTHFD}-{V}^{GT}-{V}^{AT}\\ \frac{d[CH-THF]}{dt}={V}^{MTHFD}-{V}^{MTHFC}+{V}^{MTHFS}+{V}^{GFIT/FITCD}-{V}^{5THFS2}\\ \frac{d[DHF]}{dt}={V}^{TS}-{V}^{DHFR}\\ \frac{d[5-THF]}{dt}={V}^{5THFS1}+{V}^{5THFS2}-{V}^{MTHFS}\\ \frac{d[CH3-THF]}{dt}={V}^{MTHFR}-{V}^{MS}\\ \frac{d[Formate]}{dt}={V}^{Formate}-{V}^{FTHFS}\\ \frac{d[FIGlu]}{dt}={V}^{FIGlu}-{V}^{GFIT/FITCD}\\ \frac{d[GAR]}{dt}={V}^{GAR}-{V}^{GT}\\ \frac{d[AICAR]}{dt}={V}^{GT}-{V}^{AT}\end{array}$$

Here, *V*^*X*^ is the rate of the reaction catalyzed by the enzyme X or the flux of substance X. Other designations and abbreviations are listed in the Table 1. The complete model includes the model of methionine metabolism in rodent hepatocytes, which is linked to folate metabolism via the methionine synthase reaction (Fig. 1) and via inhibition of MTHFR by AdoMet and inhibition of GNMT by CH3-THF^15^. Concentrations of ATP, ADP, NADP, NADPH, and a number of other metabolites are assumed to be constant (Supplementary text S1). The equations for the reaction rates are described in Supplementary texts S2 and S3, and the equation parameters are presented in Supplementary texts S3 and S4. The parameter values of the methionine metabolism model were not altered from the published version^15^, except for the kinetic parameters for MS and MTHFR. For these two enzymes, the kinetic parameters were adjusted to better fit experimental values obtained for rat liver enzymes using the polyglutamate forms of folates. Importantly, the activities of all enzymes in the model were obtained for rat liver or hepatocytes; enzyme activities often vary between species and tissues to a greater extent than other parameters. For other parameters, we used values obtained in different species and tissues only if data for rat liver or hepatocytes were not available in the literature. We assumed that all folates in the model had polyglutamate tails consisting of five or six glutamate residues and used enzyme parameters for their polyglutamate forms.

**Table 1:** List of abbreviations.

Enzymes/Reactions		Metabolites	
5THFS1	synthesis of 5-THF from 10-THF	5-THF	5-formyltetrahydrofolate
5THFS2	synthesis of 5-THF from CH-THF	10-THF	10-formyltetrahydrofolate
AHC	adenosine homocysteinase, EC 3.3.1.1	AdoHcy	S-adenosylhomocysteine
AT	aminoimidazolecarboxamide ribonucleotide transformylase, EC 2.1.2.3	AdoMet	S-adenosylmethionine
BHMT	betaine homocysteine methyltransferase, EC 2.1.1.5	AICAR	5-amino-1-(5-phospho-D-ribosyl)imidazole-4-carboxamide
CBS	cystathionine β-synthase, EC 4.2.1.22	CH-THF	5,10-methenyltetrahydrofolate
DHFR	dihydrofolate reductase, EC 1.5.1.3	CH2-THF	5,10-methylenetetrahydrofolate
FTHFD	formyltetrahydrofolate dehydrogenase, EC 1.5.1.6	CH3-THF	5-methyltetrahydrofolate
FTHFS	formyltetrahydrofolate synthetase, EC 6.3.4.3	DHF	dihydrofolate
GFIT/FITCD	glutamate formimidoyltransferase / formimidoyltetrahydrofolate cyclodeaminase, EC 2.1.2.5 / EC 4.3.1.4	DMG	dimethylglycine
GNMT	glycine-N-methyltransferase, EC 2.1.1.20	FAICAR	5-formamido-1-(5-phospho-D-ribosyl)imidazole-4-carboxamide
GT	glycinamide ribonucleotide transformylase, EC 2.1.2.2	FGAR	N2-formyl-N1-(5-phospho-D-ribosyl)glycinamide
MATI	methionine adenosyltransferase I, EC 2.5.1.6	FIGlu	N-formimidoyl-L-glutamate, (N-formiminoglutamate)
MATIII	methionine adenosyltransferase III, EC 2.5.1.6	GAR	N1-(5-phospho-D-ribosyl)glycinamide
METH	functional methylases	Glu	glutamate
MS	methionine synthase, EC 2.1.1.13	Gly	glycine
MTHFC	methenyltetrahydrofolate cyclohydrolase, EC 3.5.4.9	Hcy	homocysteine
MTHFD	methylenetetrahydrofolate dehydrogenase, EC 1.5.1.5	His	histidine
MTHFD/C	channeling activity of MTHFD/MTHFC	Met	methionine
MTHFR	methylenetetrahydrofolate reductase, EC 1.5.1.20	Pi	orthophosphate
MTHFS	methenyltetrahydrofolate synthetase, EC 6.3.3.2	R	acceptor of methyl groups
SHMT	serine hydroxymethyltransferase, EC 2.1.2.1	R-CH3	methylated acceptor of methyl groups
TS	thymidylate synthase, EC 2.1.1.45	Ser	serine
		THF	tetrahydrofolate

Because folate accumulation and degradation in rat hepatocytes are very slow (Supplementary text S5), we considered the folate pool (sum of concentrations of all folates involved in folate metabolism, according to the scheme in Fig. 1) as a model parameter (Table 2).

**Table 2:** Varying model parameters. Experimental range and values set in the model as the normal physiological values.

Parameter (units)	Model (normal physiological values)	Experiment	Comments and References
*V*^*Formate*^ (mmol/h/kg liver)	3.0^a^	11–20	Rat^64^.
		7.2	Rat^65^.
		4.3	Calculated using data for rat from^40^
*V*^*FIGlu*^ (mmol/h/kg liver)	0.73^b^	0.73	Rat liver. Calculated using data from^66–68^.
*V*^*GAR*^ (mmol/h/kg liver)	0.072	0.072	Rat liver^69^.
*V*^*Met*^ (mmol/h/kg liver)	0.76	0.5–1.3	Mouse liver^15^.
${V}_{max}^{TS}$ (mmol/h kg liver)	0.09	0.04–0.14	Rat liver^70–72^
Folate pool (µM)	20	5–26	^34, 44, 48, 49, 51, 66, 73– 76^

^a^Distribution of formate-consuming enzyme FTHFS in rat tissues^39^ and data obtained by *in vivo* formate infusion^40^ show that most formate produced in the rat body (about 70%) is utilized in the liver. Accordingly, we used the rate of total formate production in the rat body normalized against liver mass as a model parameter describing formate influx into liver folate metabolism (*V*^*Formate*^). For purposes of normalization, we assumed that liver mass constitutes 5% of rat body mass^41^.

^b^N-formimidoyl-L-glutamate (FIGlu) is a product of histidine catabolism^66^ and a substrate for GFIT/FITCD in folate metabolism. The rate of FIGlu production under physiological conditions can be estimated as the rate of the histidase reaction. Assuming hyperbolic dependence of histidase reaction rate on histidine concentration, and using histidase activity of 8.0 mmol/h kg liver^66,68^, Michaelis constant for histidine of 3.0 mM^77^, and histidine concentration in liver of 0.3 mM^66,67^ we obtain a rate of 0.73 mmol/h kg liver.

The main metabolic processes associated with cytoplasmic folate metabolism in mammalian cells are *de novo* purine synthesis, synthesis of dTMP from dUMP, synthesis of Met via methylation of homocysteine (Hcy) in the MS reaction, histidine catabolism, and formate utilization/production. The rates of these processes, which produce or consume one-carbon equivalents, are represented in the model by five input parameters described below.

*De novo* purine synthesis occurs via a linear chain of reactions, of which two, catalyzed by GT and AT, depend on [10-THF] and were included in the model. The rate of production of GAR, the substrate for GT reaction, is the model parameter that determines the rate of purine synthesis (*V*^*GAR*^). For simplicity, we assume that AICAR, the substrate for the AT reaction, is produced directly by the GT reaction (system of model equations (1)), and consequently skip the five intermediate reactions and metabolites between GT and AT in the pathway of *de novo* purine synthesis.

dTMP is synthesized from dUMP via the TS reaction. We assumed that the concentration of dUMP is constant and the rate of dTMP synthesis in the model is determined by TS activity (*V*^*TS*^_*max*_), which is a model parameter.

The rate of the MS reaction in liver depends on [Met], which in turn depends on Met influx from food or other sources^15^. The influx of methionine (*V*^*Met*^) is a model parameter.

FIGlu, one of the final products of histidine degradation, is the substrate for the bifunctional enzyme GFIT/FITCD in folate metabolism. The influx of FIGlu (*V*^*FIGlu*^) is a model parameter.

Formate can be produced in a number of intracellular processes, including catabolism of tryptophan, cytochrome P450-catalyzed demethylation, peroxisomal α-oxidation of phytanic acid, and degradation of glycine, serine, sarcosine, or dimethylglycine in mitochondria^38^. The distribution of the formate-consuming enzyme FTHFS in rat tissues^39^ and data obtained by *in vivo* formate infusion in rats^40^ revealed that most of the formate produced in the rat body (about 70%) is utilized in the liver. Therefore, we used the rate of total formate production in the rat body, normalized against liver mass, as the model parameter describing formate influx into liver folate metabolism (*V*^*Formate*^). For purposes of normalization, we assumed that the liver constitutes 5% of rat body mass^41^.

We varied the model input parameter values and folate pool value during the model analysis. The physiological values of these parameters are presented in Table 2.

### Calculation methods

Kinetics of the model was studied using the CVODE method for solving stiff systems of ordinary differential equation^42^. Steady-state analysis of the model was done using the AUTO software^43^. The characteristic time of each individual metabolite was computed as described under Results and discussion in the section named “*Characteristic times in folate metabolism.”*

## Results and Discussion

### Steady-state characteristics of folate metabolism

A major problem in simulation of folate metabolism is the wide scatter in the experimental data for folate concentrations, which is in turn associated with a wide scatter in the size of the folate pool. Folate pool sizes in liver of rats fed a standard diet, reported from the middle of the 1990s to the present, vary from 5 to 26 µmol per kg tissue (Table 2). In earlier publications, the scatter is even wider. Also, there are no reliable data regarding the concentrations of most individual folate species in cells and tissues, largely due to interconversion of folates during sample preparation and analysis^44–46^. Many authors have reported sums, e.g., [10-THF] + [5-THF] and [THF] + [CH2-THF], as more reliable values than the measured individual concentrations of these folates^44–46^. Also, information regarding components of the intracellular folate pool such as CH-THF and DHF is very scanty, and show only that these species do not exceed 10–16% of the intracellular folate pool^47–51^.

The model includes 6 varying parameters (Table 2). For the model, we set values for these parameters, which we here refer to as normal physiological values (Table 2) or normal physiological conditions. As well, we will consider calculated steady-state values of the model variables (metabolite concentrations) and reaction rates, corresponding to this set of parameters, as the normal physiological values (Table 3 and Table 4).

**Table 3:** Normal physiological steady-state values of the model variables.

Metabolite	Model (µM)	Experiment (µM)	References
**Folates**			
5-THF	0.016	0.6–2.9	^44, 51, 73, 74^
10-THF	5.3	2.9–5.4	^44, 73, 74^
CH-THF	0.25	0.15	^51^
CH2-THF	7.0	3.7–8.0	^44, 74^
CH3-THF	4.9	1.8–5.7	^44, 48, 49, 51, 66, 73, 74, 76^
DHF	10^−4^	0.6–0.8	^48, 49^
THF	2.6	0.8–7.7	^44, 51, 66, 74^
**Non-folates**			
AdoMet	75	50–170	^78, 79^
AdoHcy	34	3–40	^78, 79^
AICAR	0.18	<100	^80^
FIGlu	2.4	—	
Formate	58	40–150^a^	^37, 40, 64, 65, 81^
GAR	1.1	—	
Hcy	3.4	2–4	^35, 36, 58^
Met	53	20–75	^78, 79, 82^

^a^The experimental data show formate concentration in rat blood plasma. We were unable to find published experimental data regarding physiological formate concentration in rat liver. However, based on literature demonstrating that formate is rapidly and uniformly distributed between blood and other tissues^40,83^ we assumed that formate concentrations in liver and blood plasma are equal.

**Table 4:** Normal physiological steady-state rates and directions of reactions in the model.

Reaction	Rate (mmol/h kg liver)
AT	0.072
DHFR	0.014
FTHFD	0.84
FTHFS (towards 10-THF formation)	3.0
GFIT/FITCD	0.73
GT	0.072
MS	0.13
MTHFC (towards CH-THF formation)	0.052
MTHFD (towards CH2-THF formation)	0.8
MTHFD/C (towards CH2-THF formation)	2.0
MTHFR	0.13
MTHFS	0.4
SHMT (towards THF formation)	2.6
TS	0.014
5THFS1	0.013
5THFS2	0.38

**Figure 2:**
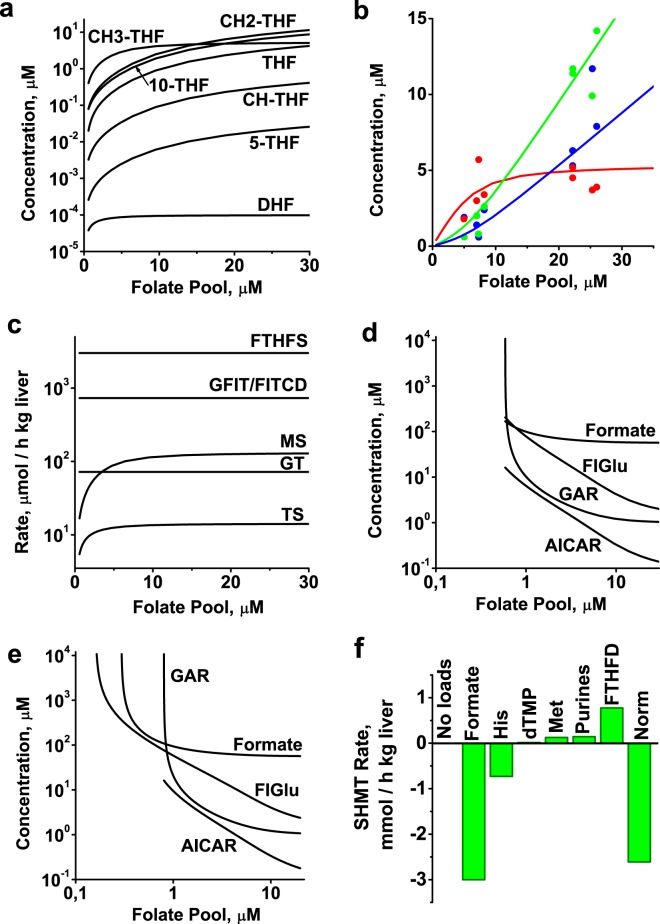
Steady-state metabolite concentrations and metabolic fluxes in folate metabolism. (**a**) Dependence of steady-state folate concentrations on folate pool size in the model. (**b**) Comparison of experimental data (symbols), showing concentrations of folates in rat liver at different folate pool sizes^44,48,49,51,66,73,74,76^, with model simulation results (lines). Red symbols and line: [CH3-THF]; green symbols and line: [THF] + [CH2-THF]; blue symbols and line: [5-THF] + [10-THF]. (**c**) Dependence of steady-state rates of FTHFS, GFIT/FITCD, MS, GT, and TS reactions on folate pool size in the model. (**d**) Dependence of steady-state concentrations of non-folate input metabolites on folate pool size in the model. (**e**) Dependence of steady-state concentration of each non-folate input metabolite on folate pool size, simulated at physiological influx of this metabolite and zero TS activity, at normal physiological methionine influx, and at zero influx of other non-folate metabolites. (**f**) Rate of SHMT reaction for different combinations of metabolic processes that produce or consume one-carbon groups. No loads: influxes of formate, FIGlu, and GAR, as well as TS and FTHFD activities, were set to zero, and methionine metabolism was disconnected from folate metabolism by setting MTHFR activity equal to zero; Formate, His, dTMP, Met, Purines, and FTHFD indicate conditions when only formate influx, histidine catabolism, dTMP synthesis, methionine metabolism, purine synthesis, or FTHFD was present in the model at normal physiological level. Norm: normal physiological steady state. Production of CH2-THF (serine consumption) is considered to be the positive direction for the SHMT reaction in the model.

Our model predicts a monotone decrease in the concentration of all folates in response to a decrease in the size of the folate pool (Fig. 2a). However, the concentrations of CH3-THF and DHF decreased much more slowly than those of other folates until the folate pool had decreased in size by more than 2-fold. The relative independence of [CH3-THF] from the folate pool stabilizes the MS reaction rate with respect to variations in folate pool near its physiological value, which is important for normal methionine recycling in methionine metabolism. This stabilization is achieved due to saturation of the CH3-THF-producing enzyme (MTHFR) by its substrate CH2-THF. As a result, the rate of production and steady-state concentration of CH3-THF do not change significantly until the decrease in the folate pool value causes [CH2-THF] to decrease well below saturating levels for MTHFR. Also, under physiological conditions, DHF is consumed in the DHFR reaction at a rate proportional to [DHF] and produced from CH2-THF in the TS reaction, which is strongly inhibited by 10-THF^52^. Because CH2-THF and 10-THF concentrations decrease along with the size of the folate pool, the DHFR rate, as well as [DHF], remain almost unchanged. Other folates, including THF, CH2-THF, 10-THF, and CH-THF, are connected via fast reversible enzymatic reactions catalyzed by MTHFD, MTHFC, MTHFD/C, and SHMT. As a result, concentrations of these folates change simultaneously, proportional to the change in the folate pool size. As mentioned above, the sums of experimentally measured concentrations [10-THF] + [5-THF] and [THF] + [CH2-THF] are more reliable than the individual concentrations of these folates due to the pronounced interconversion between them during sample preparation and analysis. The dependence of these sums and [CH3-THF] on the folate pool in the model, together with experimental data for these variables obtained in rat liver at different sizes of the folate pool, shown in (Fig. 2b), exhibit a high degree of quantitative consistency between the results of simulation and experimental data.

A significant decrease in the size of the folate pool in the model causes a decrease in the TS and MS reaction rates, whereas the rates of AT, GT, FTHFS, and GFIT/FITCD reactions remain constant (Fig. 2c). The constant rates of these reactions are the consequence of the constant influx of their corresponding non-folate substrates (GAR, formate, and FIGlu), as postulated in the model. The concentrations of these non-folate substrates increase with a decrease in the size of the folate pool (Fig. 2d) to compensate for a decrease in the concentration of the corresponding folate substrates. At a folate pool size of 0.6 µM and normal physiological influx of Met, GAR, formate, and FIGlu, along with normal physiological TS activity, the steady state in the model disappears due to unlimited accumulation of GAR (Fig. 2d).

Next, we studied the limits of the decrease in the folate pool size if the influx of only one of the non-folate substances (GAR, formate, or FIGlu) had a normal physiological value while those of the others, and the activity of TS, were equal to zero. Methionine influx cannot be equal to zero in the model because this would result in cessation of all metabolic processes due to accumulation of all folates in the form of CH3-THF. Thus, we also kept methionine influx at a normal level. Under these conditions, the steady state disappears at different folate pool sizes specific for each non-zero metabolic process associated with folate metabolism (Fig. 2e). Methionine metabolism can be disconnected from folate metabolism in the model by equating MTHFR activity to zero. In this case, folate metabolism becomes independent of methionine metabolism. If methionine metabolism is disconnected from folate metabolism while all other metabolic processes are maintained at their normal physiological rates, the steady state disappears at a folate pool size of 0.2 µM (not shown). The decrease in the minimal folate pool size in the absence of methionine metabolism in the model is explained by the fact that under these conditions, folates do not accumulate in the form of CH3-THF, and more folates can participate in metabolic processes at low folate pool sizes. If only methionine metabolism or pyrimidine synthesis remains in the model, then the folate pool can decrease to the zero. In the case of methionine metabolism, excess methionine influx is consumed via the CBS reaction (transsulfuration pathway). Thus, the minimal steady-state folate pool size depends on the cell’s metabolic state.

The existence of the steady states in the model when only one of pathways producing or consuming one-carbon groups is active reveals that folate metabolism does not require a balance between external producers and consumers of one-carbon groups associated with this metabolic system. Indeed, each of the metabolic processes associated with folate metabolism can function alone, absolutely independently of the others. Analysis of the model reveals that in this case, all necessary one-carbon groups are produced or consumed in the SHMT reaction using serine and glycine as a source or sink. This is illustrated in Fig. 2f, which shows that the rate of SHMT reaction in the model is zero if no one-carbon group producer or consumer is active. The switching on of each process that produces/provides or consumes one-carbon groups causes a corresponding change in SHMT reaction rate. Here, the FTHFD reaction was also considered as a consumer of one-carbon groups because it removes formate from metabolism in the form of CO_2_.

### Characteristic times in folate metabolism

Three characteristic times that determine the rate of response of folate metabolism to metabolic perturbations can be distinguished in this metabolic system. Changes in folate pool size associated with consumption and excretion of 5-methyltetrahydrofolate by cells, and non-catalytic degradation of folates proceeds with a characteristic time of several days (Supplementary text S5). This is very slow compared to most biochemical processes and allows us to keep the folate pool size as a parameter in our model. The characteristic time of each individual folate or other metabolite was computed at steady state by simulating an instant 3-fold increase or decrease in the metabolite’s concentration, and then determining the time during which the deviation decreased 2-fold. In addition to estimation of the characteristic times of the metabolites, this approach revealed the dynamic stability of the model. The characteristic time of [CH3-THF] is on the order of several minutes, whereas the concentrations of other folates change with characteristic times of less than one second (Table 5). These small characteristic times, from fractions of a second to minutes, enable the fast response of folate metabolism to variations in the rate of associated metabolic processes, which in turn respond to cellular demands.

**Table 5:** Characteristic times of model variables.

Folates		Non-folates	
Variable	Characteristic time (sec)	Variable	Characteristic time (sec)
[5-THF]	2∙10^−1^	[AdoHcy]	1.2∙10^2^
[10-CHO-THF]	2∙10^−1^	[AdoMet]	2∙10^3^
[CH-THF]	2∙10^−2^	[AICAR]	13
[CH2-THF]	2∙10^−1^	[FIGlu]	20
[CH3-THF]	2∙10^2^	[Formate]	10
[DHF]	3∙10^−2^	[GAR]	100
[THF]	5∙10^−2^	[Hcy]	1.2∙10^2^
		[Met]	2∙10^2^

### Independent functioning of metabolic processes associated with folate metabolism

**Figure 3:**
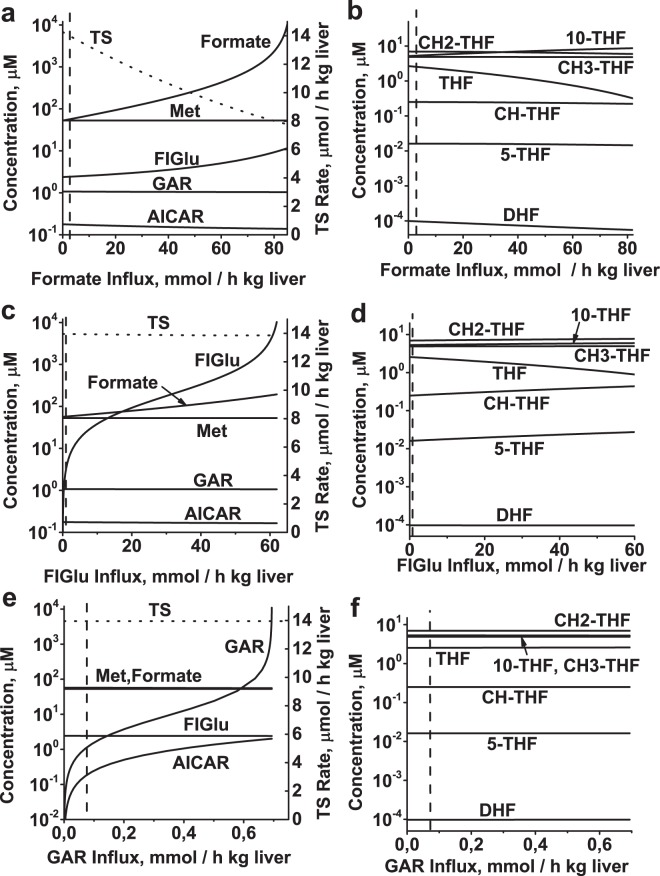
Effect of formate influx, rate of histidine catabolism, and rate of purine synthesis on steady-state metabolite concentrations and TS reaction rate in folate metabolism. Steady-state concentrations of non-folate metabolites and TS reaction rate (dotted line) and steady-state concentrations of folates as functions of formate influx (**a**,**b**), FIGlu influx **(c**,**d**), and GAR influx (**e**,**f**). Each parameter was varied while holding other model parameters at their normal physiological values. Vertical dashed lines indicate the normal physiological values of the varied parameters.

**Figure 4:**
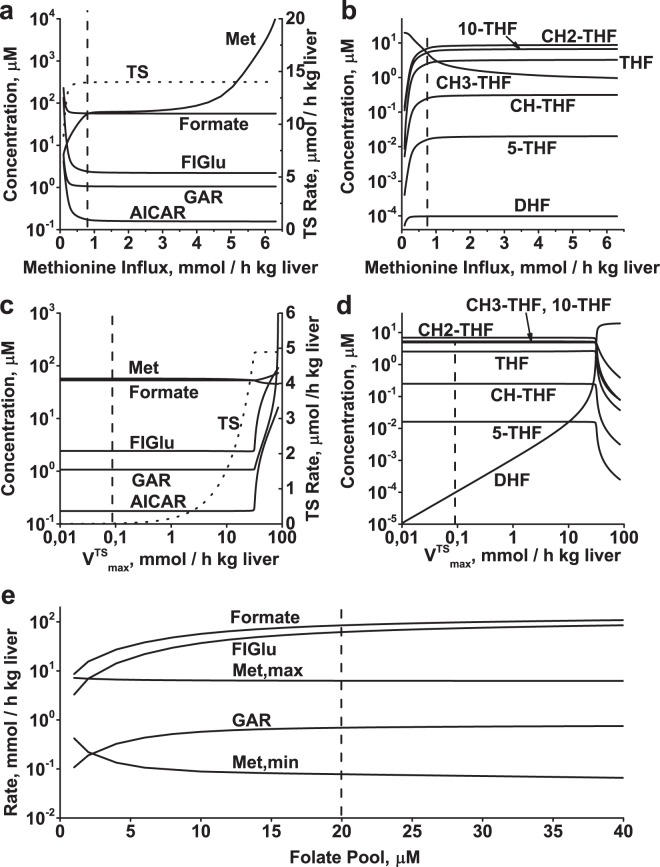
Effect of methionine influx and TS activity on steady-state metabolite concentrations and TS reaction rate in folate metabolism, and dependence of variation limits of metabolic pathways associated with folate metabolism on folate pool size. Steady-state concentrations of non-folate metabolites and TS reaction rate (dotted line) and steady-state concentrations of folates as functions of methionine influx (**a**,**b**) and TS activity (**c**,**d**). Each parameter was varied while holding other model parameters at their normal physiological values. (**e**) Dependence of maximal allowable influx of formate, FIGlu, Met, and GAR, as well as minimal influx of Met, in the model on folate pool value. Vertical dashed lines indicate normal physiological values of varying model parameters.

We studied the effect of variation in the rate of purine synthesis, histidine catabolism, formate utilization, or methionine metabolism on the steady state in folate metabolism by varying influx of GAR, FIGlu, formate, or methionine in the model. In addition, we examined the effect of variation in the rate of pyrimidine synthesis by varying TS activity. Variation of each model input parameter was performed while holding the other parameters at their physiological values. Increases in formate, FIGlu, GAR, or methionine influx above their physiological value causes a monotone increase in the steady-state level of the corresponding non-folate metabolites (formate, FIGlu, GAR, or Met) (Figs 3, 4). In the case of TS activation, a significant increase in the levels of GAR, AICAR, and FIGlu is observed only at greater than 100-fold activation of the enzyme (Fig. 4c). In this study, each input parameter was increased until the steady-state concentration of any metabolite reached value of 10 mM. We assume that accumulation of any metabolite to such level would cause metabolic and osmotic problems within the cell, and considered the corresponding input parameter value as the upper limit of activation.

Figure 3a shows that at a greater than 20-fold increase in formate influx in the model, concentrations of input metabolites in other metabolic processes, as well as the TS reaction rate, do not change dramatically: the concentration of FIGlu increases 4–5-fold, whereas the changes in other input metabolite concentrations and the TS reaction rate are much smaller. Changes in folate concentrations are small as well, and with the exception of [THF], actually negligible (Fig. 3b).

Activation of histidine catabolism (increase in FIGlu influx) up to about 80-fold affects the concentrations of input metabolites involved in other metabolic processes, as well as the TS reaction rate and folate concentrations, even less than activation of formate influx (Fig. 3c,d).

Similarly, a significant increase in GAR influx (activation of purine synthesis) or in methionine influx does not affect other metabolic processes associated with folate metabolism (Figs 3e,f, 4a,b). Interestingly, a decrease in methionine influx below its physiological level causes a significant disturbance in folate metabolism (Fig. 4a,b). This is because a decrease in methionine influx results in a decrease in the MS reaction rate, which leads in turn to accumulation of folates in the form of CH3-THF and depletion of other folate metabolites. Finally, at a methionine influx of 78 µmol/h kg liver, the steady state of folate metabolism disappears due to unlimited accumulation of formate.

Activation of pyrimidine synthesis in the model was simulated by increasing TS activity (*V*^*TS*^*max*). An increase in TS activity causes a corresponding increase in the TS reaction rate until it becomes equal to the maximal rate of the DHFR reaction (Fig. 4c). This happens at greater than 100-fold activation of dTMP synthesis in the model. Under those conditions, the DHFR reaction becomes the rate-limiting step in the pyrimidine synthesis pathway, and a further increase in TS activity causes accumulation of DHF and depletion of other folates (Fig. 4d), which significantly affects metabolic processes associated with folate metabolism (Fig. 4c) and ultimately leads to disappearance of the steady state in the model.

In the case of activation of any metabolic process associated with folate metabolism except methionine metabolism, the metabolites of methionine metabolism (AdoMet, AdoHcy, and Hcy) remain unaffected (not shown). In the case of an increase in methionine influx, concentrations of intermediates of methionine metabolism change according to predictions of a published model of methionine metabolism^15^.

The upper limits for activation of formate, FIGlu, or GAR influx decrease with the size of the folate pool (Fig. 4e). The upper limit for activation of methionine influx is almost independent of the folate pool size, whereas the lower bound on methionine influx increases as the folate pool decreases (Fig. 4e). As mentioned above, a decrease in methionine influx below its physiological level results in accumulation of folates in the form of CH3-THF and depletion of other folate metabolites, ultimately causes the steady state to disappear. Our results reveal that at low folate pool sizes, folate metabolism is more sensitive to a decrease in methionine influx below its normal physiological value.

**Figure 5:**
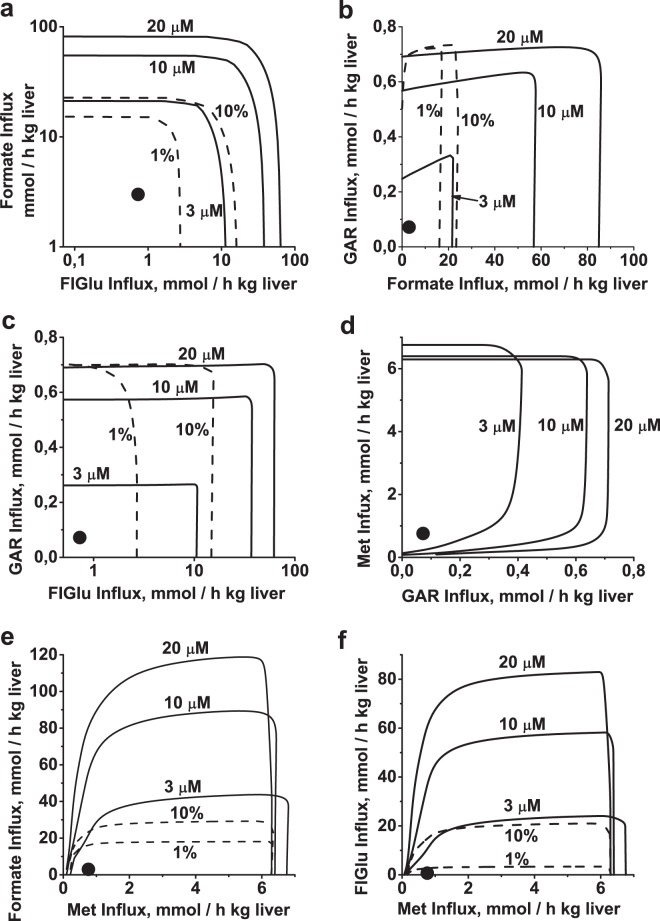
Independent functioning of metabolic processes associated with folate metabolism. Solid lines show maximum allowable rates of metabolic processes at different folate pool sizes for the following couples: (**a**) Histidine catabolism and formate utilization; (**b**) Formate utilization and purine synthesis; (**c**) Histidine catabolism and purine synthesis; (**d**) Purine synthesis and methionine metabolism; (**e**) Methionine metabolism and formate utilization; (**f**) Methionine metabolism and histidine catabolism. In each panel, non-varied parameters were held at their normal physiological levels. Folate pool sizes (in µM) are indicated next to the corresponding solid lines. Dashed lines show the maximum allowable rates of metabolic processes, obtained at a folate pool size of 20 µM and with the activities of SHMT, MTHFC, and MTHFD reduced to 10% and 1% of their initial values, as indicated. The activities of all three enzymes were decreased simultaneously. In panel d, lines obtained at 10% and 1% of initial enzymatic activity coincide with the line obtained at initial enzymatic activity. Black circle indicates normal physiological state.

Thus, our data show that metabolic processes associated with folate metabolism have large activation capacities, and that any one of them can be activated or inhibited more or less independently from the others. Moreover, the rate of any of these processes can be increased to its upper limit without significantly affecting the maximal rate of any other process. Figure 5 shows the areas of allowable metabolic fluxes for different couples of metabolic processes associated with folate metabolism. Interestingly, for the metabolic couple including formate utilization and purine synthesis, activation of one processes increases the maximal activity for the other (Fig. 5b). This can be explained by the fact that in this metabolic couple formate utilization process produces the folate substrate for purine synthesis (Fig. 1). A similar but less pronounced effect is observed for the couple of histidine catabolism and purine synthesis (Fig. 5c). Also, an increase in methionine influx causes an increase in activation limits for other metabolic processes due to elevation of the levels of their folate substrates (10-THF, CH2-THF, and THF) (Fig. 4b, Fig. 5d–f). The areas of allowable metabolic fluxes shrink as the folate pool decreases in size, but independence of activation does not disappear (Fig. 5).

The mechanism of independent functioning of different metabolic processes in folate metabolism is based on a group of highly active enzymes (SHMT, MTHFD, and MTHFC) that catalyze fast reversible reactions. These enzymes maintain the distribution of folate metabolites close to equilibrium during various perturbations in folate metabolism, thus providing stable conditions for all metabolic processes associated with this metabolic system. In general a decrease in activity of these enzymes causes a decrease in the upper limits of activation of metabolic processes associated with folate metabolism (Fig. 5 – dashed lines). This effect, however, is negligible for relatively slow metabolic processes associated with folate metabolism, such as purine synthesis and methionine production from Hcy (Fig. 5).

### Formate Turnover

**Figure 6:**
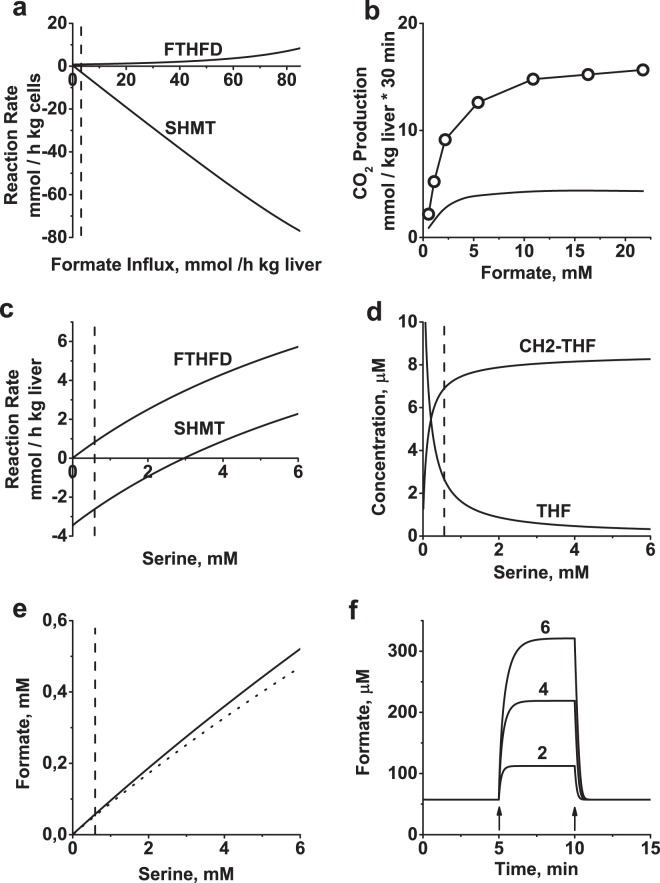
Formate turnover in liver cytoplasmic folate metabolism. (**a**) Dependence of SHMT and FTHFD reaction rates on formate influx. (**b**) Rate of CO_2_ production in rat at high formate concentrations. Symbols – experimental data obtained after injection of rats with [^14^C] formate, followed by measurement of released ^14^CO_2_^55^. Original experimental data are expressed as the rate of formate oxidation per kg of rat body weight. To compare the experimental and theoretical results, we recalculated the experimental rates as per kg of liver, assuming that all production of CO_2_ from formate occurs in the liver and that liver constitutes 5% of rat body mass. Continuous line – result of model simulation excluding formate influx, using formate concentration as a parameter. (**c**) Dependence of SHMT and FTHFD reaction rates in the model on serine concentration. Production of CH2-THF (serine consumption) is considered to be the positive direction for the SHMT reaction in the model. (**d**) Dependence of [THF] and [CH2-THF] on serine concentration in the model. (**e**) Concentration of formate as a function of serine concentration. Solid line: complete model; dotted line: model without formate influx. (**f**) Kinetics of formate concentration in the model in response to an instant increase in the serine concentration to 2, 4, and 6 times above the physiological level (to final levels of 1.18, 2.36, and 3.56 mM), followed by an instant decrease to the physiological serine level at the time points indicated by the arrows. Vertical dashed lines in panels a, c, d, and e indicate the normal physiological concentration of serine.

In mammalian cells, formate, which is produced in a number of metabolic processes (predominantly glycine degradation via glycine cleavage system in mitochondria), acts as the main source of one-carbon groups for folate metabolism in the cytoplasm^38^. Our estimations show that in rat liver under physiological conditions, utilization of formate is the most intensive metabolic process associated with cytoplasmic folate metabolism (Tables 2, 4, Fig. 2c,f). The total rate of two metabolic fluxes providing one-carbon groups for folate metabolism (formate production and histidine catabolism) is equal to 3.73 mmol/h kg liver, with format accounting for ∼80% of the total. This is almost 13-fold higher than the total liver demand for one-carbon equivalents for synthesis of purines, pyrimidines, and Hcy methylation (Tables 2, 4 Fig. 2c,f). Toxicokinetic modeling of formate elimination in rats has shown that most formate in the rat body is utilized in the liver^40^, and this is consistent with the distribution of FTHFS activity in rat tissues^39^. Thus, one can speculate that just a fraction of the formate produced in non-liver tissues may be used as a one-carbon source to support purine and pyrimidine synthesis and Hcy methylation in these tissues, while the rest is recycled or eliminated in the liver. Interestingly, significant formate overproduction has been observed in a number of cultured human non-liver cell lines^53,54^. Our model shows that under physiological conditions, most of the one-carbon groups (∼70%) entering folate metabolism in rat liver are converted to serine, whereas a small fraction (∼23%) is eliminated as CO_2_ via the FTHFD reaction (Fig. 6a). The amount and fraction of one-carbon groups eliminated by FTHFD increase as the formate influx/concentration increases (Fig. 6a). Activation of formate oxidation to CO_2_ at high formate concentrations is consistent with the results of *in vivo* experiments (Fig. 6b)^55^.

In addition, the model predicts an increase in one-carbon group disposal via FTHFD when the SHMT reaction equilibrium is shifted towards less serine consumption by increasing serine levels (Fig. 6c). An increase in serine concentration causes accumulation of CH2-THF and a decrease in the concentration of THF (Fig. 6d), which is a strong inhibitor of FTHFD. As a result, the FTHFD reaction rate increases. Additionally, an increase in serine levels causes an increase in the steady-state concentration of formate (Fig. 6e) because the increase in CH2-THF levels shifts the equilibrium in the MTHFD/C reactions to higher 10-THF levels, which in turn shifts the equilibrium in the FTHFS reaction to higher formate levels. This mechanism can provide physiological steady-state formate levels in the model, even in the absence of an external formate influx (Fig. 6e). Accumulation of formate after addition of serine has been observed experimentally in several cell lines^53^. Our results demonstrate that at least in rat liver, cytoplasmic folate metabolism can maintain significant formate levels in the absence of folate influx from mitochondria or other tissues. In the model, accumulation of formate after an increase in serine levels is fast and reversible (Fig. 6f). As mentioned above, under normal physiological conditions, the SHMT reaction is directed towards serine production (Fig. 6c). The rate of serine production in the SHMT reaction decreases as the serine concentration increases, and the reactions switch to serine consumption at serine concentration of 3 mM (Fig. 6c), corresponding to [Gly]/[Ser] = 0.6 (versus about 3.0 at normal steady state).

### Folate Pool and Methionine Metabolism

**Figure 7:**
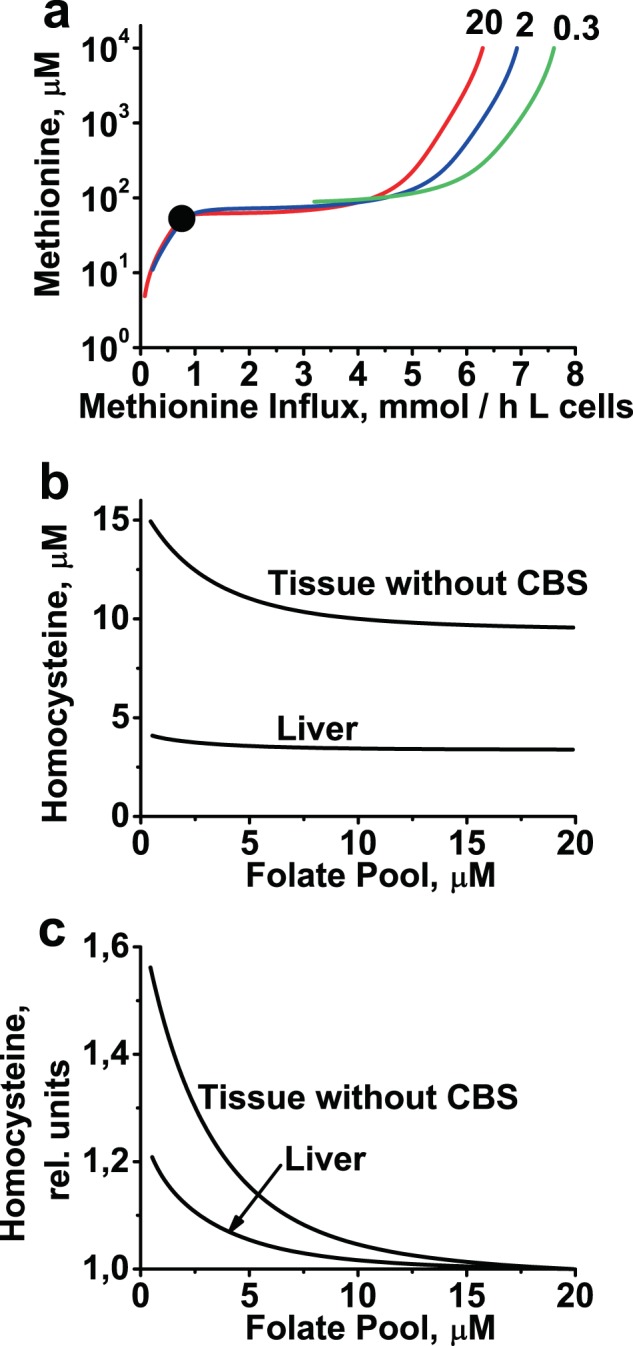
Effects of folate pool on methionine metabolism. (**a**) Dependence of methionine concentration on methionine influx at folate pool sizes of 0.3, 2, and 20 µM, as indicated by the numbers above the curves. The curves remain almost unchanged at folate pool sizes from 20 to 200 µM. Black circle indicates position of normal physiological steady state in the model. (**b**) Steady-state homocysteine levels in liver and non-liver tissue lacking transsulfuration pathway, as a function of folate pool size; (**c**) the same data as in panel b, after normalization of each curve to [Hcy] obtained at a folate pool size of 20 µM.

Folate metabolism is tightly linked to methionine metabolism via the methionine synthase reaction, as well as allosteric inhibition of GNMT by CH3-THF and MTHFR by AdoMet^27^. Some aspects of the interaction of methionine metabolism with folate metabolism were described above. One of the significant features of methionine metabolism in liver is stabilization of methionine concentration with respect to variation in methionine influx^56^. The model shows that a decrease in folate pool size causes a decrease in stabilization area due to the disappearance of steady states at low methionine influx (Figs 3f, 7a). Increasing the folate pool size up to 10 times above its physiological level does not affect stabilization of methionine concentration (not shown).

One of the negative consequences of folate deficiency *in vivo* is significant elevation of blood plasma Hcy levels^33–37^. Hcy is an intermediate in methionine metabolism (Fig. 1), and elevated Hcy levels in blood plasma are associated with cardiovascular diseases and other pathologies^5,57^. Interestingly, the concentration of Hcy is lower in the liver than in plasma^35,36,58,59^, and in the case of folate deficiency, liver [Hcy] increases much less than plasma [Hcy]^35,36^. Liver cells have a highly active transsulfuration pathway that can efficiently clear intracellular Hcy. Together, these observations suggest that plasma Hcy levels are determined by non-liver tissues that lack the transsulfuration pathway, such as skeletal and heart muscles. To test this hypothesis, we modified the model by removing CBS and liver-specific enzymes MAT3 and GNMT. To compare liver cells with cells lacking the transsulfuration pathway, we must use methionine concentration as a model parameter instead of assuming a constant input flow of methionine. This is necessary because in the absence of the transsulfuration pathway the modified model cannot provide a steady state at a constant input flow of methionine. Thus, we used a constant methionine concentration equal to 53 µM (normal physiological methionine concentration, Table 3) as a model parameter. The modified model has a physiological Hcy concentration of 9.6 µM, that is about 2.8-fold higher than the level in the liver (Table 3, Fig. 7b). Moreover, the modified model predicts a ~56% increase in Hcy levels when the folate pool size decreases, whereas for liver cells our model predicts only a 21% increase in Hcy levels under the same conditions (Fig. 7c). In this case, the increase in Hcy levels is a result of the decrease in MS reaction rate due to the decrease in folate levels. However, in cells with an active transsulfuration pathway, such as hepatocytes, an increase in Hcy levels causes an increase in the CBS reaction rate, providing an additional sink for Hcy and diminishing the elevation of [Hcy] relative to cells that lack this pathway. Thus, the simulation results strongly suggest that the increase in plasma Hcy levels observed in cases of folate deficiency^35,36^ is due to tissues other than liver.

## Conclusions

Our mathematical model provides a good quantitative fit for various experimental data sets previously obtained for different forms of folates in rat liver (Fig. 2b). The model suggests that folate metabolism has a steady state for a wide range of folate pool values. However, at a very low folate pool values (below 1 µM) folate metabolism cannot provide adequate rates of utilization of the input metabolites. This leads to disruption of the steady state and unlimited accumulation of input metabolites of the metabolic pathways associated with folate metabolism (Fig. 2d,e).

**Figure 8:**
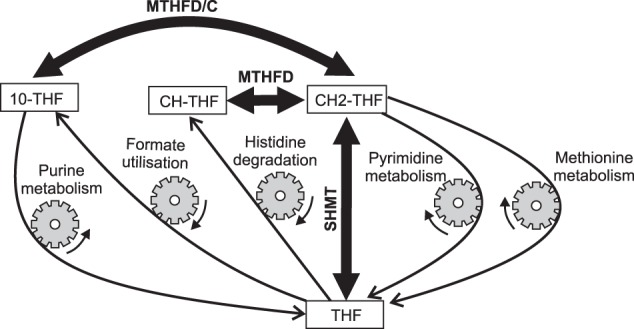
Mechanism underlying independent functioning of metabolic processes associated with folate metabolism. Thin arrows show metabolic fluxes in formate metabolism, histidine catabolism, purine synthesis, dTMP synthesis, and methionine metabolism. Bold arrows show fast reversible reactions catalyzed by SHMT, MTHFC, and MTHFD.

The most interesting prediction of our model is the relative independence of metabolic processes associated with folate metabolism (Figs 3–5). The model predicts that each of these processes (i.e., formate utilization, histidine catabolism, purine synthesis, dTMP synthesis, and methionine recycling) can be activated or suppressed independently of the others, in accordance with cellular demands. This independence is provided by a group of highly active enzymes (SHMT, MTHFD, and MTHFC) that catalyze very fast equilibrium reactions (Fig. 8). These reactions maintain the ratios between the main folate substrates and folate products of processes associated with folate metabolism (i.e. THF, CH2-THF, CH-THF, and 10-CHO-THF) almost independent of the rates of these processes. Thus, each metabolic process associated with folate metabolism actually depends almost exclusively on its input (non-folate) parameter (e.g., formate input flow, methionine input flow, TS activity etc.), which in turn depends on cellular demand for the corresponding process. In this way, folate metabolism provides cells with the possibility of functioning in a multitask mode, with independent activation or suppression of different processes associated with this metabolic system.

Our results reveal that rat liver folate metabolism represents a typical case of modular organization of cellular metabolism^60,61^ with several separate metabolic systems (purine metabolism, pyrimidine metabolism, methionine metabolism, etc) connected via folates and functioning almost independent of each other. Interestingly, the degree of independence between the systems can be different at the same metabolic network topology. Indeed, Fig. 5 shows that a decrease in folate pool value as well as a decrease in activity of SHMT, MTHFC, and MTHFD, causes a significant decrease in the area of independent functioning of the metabolic systems associated with folate metabolism. It can be expected that the main condition for the independence of the associated processes in folate metabolism is the high activity of enzymes redistributing different folate forms between the associated processes compared with metabolic fluxes in the associated processes. In other words, the characteristic times of folates should be small compared with the characteristic times of metabolites in the associated metabolic processes.

Quantitative analysis of the metabolic fluxes associated with folate metabolism in rat liver reveals that rat tissues produce a significant excess of formate relative to the demand for one-carbon groups. As a result, most of the formate entering folate metabolism in the rat liver is recycled to serine or eliminated as CO_2_ via FTHFD. Accordingly, it is reasonable to assume that the majority of formate produced in cells is not determined by the need to fuel folate metabolism, but is instead associated with other cellular needs such as regulation of glycine levels via the glycine cleavage system in mitochondria. This assumption is supported by the results of our model, which demonstrate that cytoplasmic folate metabolism in the liver can maintain significant formate levels even in the absence of formate influx from external sources such as mitochondria or other tissues (Fig. 6e).

Another interesting finding of our model is that a decrease in folate pool size should cause a much larger elevation of Hcy in cells lacking the transsulfuration pathway than in hepatocytes (Fig. 7b,c). Therefore, cardiovascular tissues lacking the transsulfuration pathway, such as heart muscle and endothelium may be more vulnerable to deficiency in folates because under such conditions these tissues may be exposed to the highest intracellular Hcy concentrations. Moreover, one may suggest that tissues other than the liver are responsible for high blood Hcy levels in cases of folate deficiency.

Finally, we wish to emphasize that while our model, like some other models, describes the dependence of folate metabolism on the size of the folate pool, to our knowledge no model to date has described regulation of folate pool size in mammalian cells. The size of the intracellular folate pool seems to depend on folate availability in food and folate levels in blood. It is not clear, however, whether the folate pool passively follows blood folate levels or is actively maintained at some optimal size when folates are available in excess. Similar questions can be raised regarding pools of other metabolic cofactors such as adenylates, glutathione, NAD(H), etc. In previous work, we obtained strong evidence that adenylate and glutathione pools are determined by the rate of ATP and reduced glutathione turnover in the cell, respectively^62,63^. Variations in the size of cofactor pools, including the folate pool, could provide an additional level of metabolic regulation that merits further investigation.

## Supplementary information

Supplementary information Rat liver folate metabolism can provide an independent functioning of associated metabolic pathways

## Data Availability

All data generated or analyzed during this study are included in this article (and its Supplementary Information Files).
