# Supplementary material for: Rat liver folate metabolism can provide an independent functioning of associated metabolic pathways
Source: Sci Rep. 2019 May 21;9:7657. doi: 10.1038/s41598-019-44009-5 (PMC6529478; doi:10.1038/s41598-019-44009-5)
Supplement: Supplementary file 1 — Supplementary information Rat liver folate metabolism can provide an independent functioning of associated metabolic pathways [file 41598_2019_44009_MOESM1_ESM.pdf]

Supplementary information

Rat liver folate metabolism can provide an independent functioning of associated metabolic pathways

Aleksandr V. Zaitsev[1], Michael V. Martinov[2], Victor M. Vitvitsky[2], Fazoil I. Ataullakhanov[1,2,3].

[1]Department of Physics, Moscow State University, Moscow 119991, Russia, [2]Center for Theoretical Problems of Physico-Chemical Pharmacology, Russian Academy of Sciences, Moscow 119991, Russia, [3]Dmitry Rogachev National Medical Research Center for Pediatric Hematology, Oncology, and Immunology, Moscow 117997, Russia.

Supplementary text S1. Constant metabolite concentrations in the model.

Table S1.1. Constant metabolite concentrations in the model.

**Metabolite**	**Model (μM)**	**Experiment (μmol/kg liver)**	**References**
ATP	1600	1500 - 2000	1–4
ADP	1000	600 - 1300	1,5,6
dUMP	200	170 - 240	7
Gly	1800	1400 – 1900	8–10
NADP	15	4-54	11–13
NADPH	320	180 - 370	11–13
Pi	2300	2100 - 2700	1
Ser	590	400 - 700	8–10

References

1. Kaminsky, Y. G., Kosenko, E. A. & Kondrashova, M. N. Analysis of the circadian rhythm in energy metabolism of rat liver. *Int. J. Biochem.* **16,** 629–639 (1984).

2. Forslund, A. H. *et al.* The 24-h whole body leucine and urea kinetics at normal and high protein intakes with exercise in healthy adults. *Am J Physiol* **275,** E310-20 (1998).

3. Wiechetek, M., Breves, G. & Holler, H. Effects of increased blood ammonia concentrations on the concentrations of some metabolites in rat tissues. *Q.J.Exp.Physiol*

423–429 (1981). doi:10.1113/expphysiol.1981.sp002584

4. Chen, L. *et al.* Impaired liver regeneration in mice lacking methionine adenosyltransferase 1A. *FASEB J.* **18,** 914–916 (2004).

5. Harvey, P. J., Gready, J. E., Yin, Z., Le Couteur, D. G. & McLean, a J. Acute oxygen supplementation restores markers of hepatocyte energy status and hypoxia in cirrhotic rats. *J. Pharmacol. Exp. Ther.* **293,** 641–5 (2000).

6. Kaminsky, Y. G., Kosenko, E. A. & Kondrashova, M. N. Alteration of adenine nucleotide pool in old rat liver and its normalization with ammonium succinate. *FEBS Lett.* **159,** 259–261 (1983).

7. Pels Rijcken, W. R., Hooghwinkel, G. J. & Ferwerda, W. Pyrimidine metabolism and sugar nucleotide synthesis in rat liver. *Biochem. J.* **266,** 777–783 (1990).

8. Jacobs, R. L., Stead, L. M., Brosnan, M. E. & Brosnan, J. T. Hyperglucagonemia in rats results in decreased plasma homocysteine and increased flux through the transsulfuration pathway in liver. *J.Biol.Chem.* **276,** 43740–43747 (2001).

9. Adibi, S. A. Interrelationships between level of amino acids in plasma and tissues during starvation. *Am J Physiol* **221,** 829–838 (1971).

10. Scheer, J. B., Mackey, A. D., Gregory III, J. F. & Gregory, J. F. Activities of hepatic cytosolic and mitochondrial forms of serine hydroxymethyltransferase and hepatic glycine concentration are affected by vitamin B-6 intake in rats. *J. Nutr.* **135,** 233–238 (2005).

11. Clark, J. B. & Pinder, S. Control of the steady-state concentrations of the nicotinamide nucleotides in rat liver. *Biochem. J.* **114,** 321–330 (1969).

12. Lazzarino, G. *et al.* Single-sample preparation for simultaneous cellular redox and energy state determination. *Anal. Biochem.* **322,** 51–59 (2003).

13. Tischler, M. E., Friedrichs, D., Coll, K. & Williamson, J. R. Pyridine nucleotide distributions and enzyme mass action ratios in hepatocytes from fed and starved rats. *Arch. Biochem. Biophys.* **184,** 222–236 (1977).

Supplementary text S2. Reaction rate equations.

AT (AICAR Transformylase, EC 2.1.2.3). AT catalyzes irreversible reaction of formyl group transfer from 10-THF to AICAR via a random sequential bi-bi mechanism [1] providing the second of the two folate-requiring reactions in purine *de novo* biosynthesis. The reaction rate in the model is described by the corresponding equation:

$$V^{AT} = V_{max}^{AT} \frac{[AICAR]}{[AICAR]+K_{m,AICAR}^{AT}} \cdot \frac{[10-THF]}{[10-THF]+K_{m,10-THF}^{AT}} \quad \text{(S2.1)}$$

DHFR (Dihydrofolate reductase, EC 1.5.1.3). DHFR catalyzes irreversible reduction of DHF to THF using NADPH as a cofactor. The reaction mechanism is random sequential bi-bi [2]. Under physiological conditions the enzyme is saturated with NADPH (Supplementary texts S1, S4). Thus, the reaction rate can be described by Michaelis-Menten equation with one substrate:

$$V^{DHFR} = V_{max}^{DHFR} \frac{[DHF]}{K_m^{DHFR}+[DHF]} \quad \text{(S2.2)}$$

FTHFD (Formyltetrahydrofolate dehydrogenase, EC 1.5.1.6). FTHFD catalyzes irreversible reaction of NADP - dependent oxidation of 10-THF to THF and CO_2 [3,4]. Under intracellular conditions the enzyme is supposed to be saturated with NADP, since the corresponding Michaelis constant value is substantially lower than the intracellular NADP concentration (Supplementary texts S1, S4). Also, the enzyme is strongly inhibited by THF [5–7]. In the model the FTHFD reaction rate is described by the equation taken from [7]:

$$V^{FTHFD} = \frac{V_{\max}^{FTHFD}[10-THF]}{K_{m,10-THF}^{FTHFD}\left(1+\frac{[THF]}{K_{i,THF}^{FTHFD}}\right)+[10-THF]} \quad \text{(S2.3)}$$

FTHFS (Formyltetrahydrofolate synthetase, EC 6.3.4.3). FTHFS catalyzes the reversible ATP-dependent synthesis of 10-formyltetrahydrofolate from formate and tetrahydrofolate. While a ping-pong mechanism was proposed for the reaction based on the enzyme crystal structure analysis [8] there are no strong evidences supporting this mechanism. The reaction rate in the model is described by equation for random-order sequential bi-bi mechanism [9,10] with assumption that enzyme is saturated with ATP and ADP (Supplementary texts S1, S4).

$$V^{FTHFS} = \frac{\frac{V_{max}^{FTHFS}}{K_{m,THF}K_{m,HCOOH}}\left([THF][HCOOH] - \frac{[10-THF][P_i]}{K_{eq}^{FTHFS}\frac{[ATP]}{[ADP]}}\right)}{1 + \frac{[THF]}{K_{m,THF}^{FTHFS}} + \frac{[HCOOH]}{K_{m,HCOOH}^{FTHFS}} + \frac{[10-THF]}{K_{m,10-THF}^{FTHFS}} + \frac{[P_i]}{K_{m,Pi}^{FTHFS}} + \frac{[THF][HCOOH]}{K_{m,THF}^{FTHFS}K_{m,HCOOH}^{FTHFS}} + \frac{[10-THF][P_i]}{K_{m,10-THF}^{FTHFS}\,K_{m,Pi}^{FTHFS}}}$$

(S2.4)

GFIT/FITCD (Glutamate formimidoyltransferase/formimidoyltetrahydrofolate cyclodeaminase, EC 2.1.2.5/EC 4.3.1.4) GFIT/FITCD is a bifunctional enzyme that catalyzes two consecutive reactions: transfer of formimino group from formiminoglutamate to tetrahydrofolate, and deamination of formiminotetrahydrofolate with formation of CH-THF. Transferase reaction follows the random sequential bi-bi mechanism [11]. The overall reaction is considered to be irreversible [12,13]. For the polyglutamate forms of folates, almost 100% channeling between the catalytic sites of the enzyme was shown [14]. Therefore, we did not include in the model formiminotetrahydrofolate and describe the rate of the overall reaction using a single equation for random sequential bi-bi mechanism:

$$V^{GFIT/FITCD} = V_{max}^{GFIT/FITCD}\frac{[FIGlu]}{[FIGlu]+K_{m,FIGlu}^{GFIT/FITCD}} \cdot \frac{[THF]}{[THF]+K_{m,THF}^{GFIT/FITCD}} \quad \text{(S2.5)}$$

GT (GAR Transformylase, EC 2.1.2.2). This enzyme catalyzes the irreversible transfer of the formyl group from 10-formyltetrahydrofolate to the amino nitrogen of GAR [15], providing the first of the two folate-requiring reactions in purine *de novo* biosynthesis. According to the literature data [16] the reaction rate is described by equation for ordered sequential (bi-bi) mechanism with 10-THF binding first:

$$V^{GT} = V_{max}^{GT}\frac{[GAR][10-THF]}{K_{i,10-THF}^{GT}\,K_{m,GAR}^{GT} + K_{m,GAR}^{GT}[10-THF] + K_{m,10-THF}^{GT}[GAR] + [10-THF][GAR]}$$

(S2.6)

MS (Methionine synthase, EC 2.1.1.13). This enzyme catalyzes an irreversible methyl group transfer from CH3-THF to homocysteine via an ordered bi-bi mechanism with CH3-THF as the first substrate and methionine as the first product [17,18]. The equation for MS reaction rate was taken from [19]:

$$V^{MS} = \frac{V_{max}^{MS}}{1+\frac{K_{m,CH3-THF}^{MS}}{[CH3-THF]}+\frac{K_{m,Hcy}^{MS}}{[Hcy]}\left(1+\frac{K_{d,CH3-THF}^{MS}}{[CH3-THF]}\right)} \quad (S2.7)$$

We have changed MS activity in the model from 6.6 [19] to 3 mmol/h kg liver for better description of experimental dependence of CH3-THF concentration on folate pool.

MTHFD/MTHFC (Methylentetrahydrofolate dehydrogenase / Methylenetetrahydrofolate cyclohydrolase, EC 1.5.1.5 / EC 3.5.4.9). MTHFD and MTHFC reactions are catalyzed by the same trifunctional cytoplasmic enzyme – C1-tetrahydrofolate-synthase (C1-THFS). MTHFD activity provides the reversible NADP/NADPH dependent transformation between CH2-THF and CH-THF. MTHFC provides the reversible transformation between 10-THF and CH-THF. Also, the enzyme demonstrates effective channeling between MTHFD and MTHFC. Equations for corresponding reaction rates were constructed in this work and described in Supplementary text S3 together with the enzyme kinetic parameters.

MTHFR (Methylenetetrahydrofolate reductase, EC 1.5.1.20). This enzyme catalyzes irreversible reduction of CH2-THF to CH3-THF using NADPH as a cofactor. The reaction mechanism is ping-pong with NADPH as the first substrate and CH3-THF as the second product. MTHFR is allosterically inhibited by AdoMet. The equation for MTHFR reaction rate was taken from [19].

$$V^{MTHFR} = V_{max}^{MTHFR} \frac{\frac{1}{1+\frac{[AdoMet]}{K_{i,AdoMet}^{MTHFR}\left(1+\frac{[AdoHcy]}{K_{i,AdoHcy}^{MTHFR}}\right)}}+\frac{0.046}{1+\frac{K_{i,AdoMet}^{MTHFR}\left(1+\frac{[AdoHcy]}{K_{i,AdoHcy}^{MTHFR}}\right)}{[AdoMet]}}}{1+\frac{K_{m,NADPH}^{MTHFR}}{[NADPH]}\left(1+\frac{[CH_3-THF]}{K_{i,CH3-THF}^{MTHFR}}\right)+\frac{K_{m,CH2-THF}^{MTHFR}}{[CH_2-THF]}} \quad (S2.8)$$

Activity of MTHFR in the model was changed from 4 [19] to 0.9 mmol/h kg liver for better description of experimental dependence of CH3-THF concentration on folate pool.

MTHFS (Methenyltetrahydrofolate synthase, EC 6.3.3.2). This enzyme catalyzes irreversible ATP-dependent conversion of 5-THF to CH-THF [20,21]. Reaction mechanism is random sequential bi-bi [22]. In cells the enzyme is saturated with ATP (Supplementary texts S1, S4), so the reaction rate is described by the Michaelis-Menten equation with only one substrate:

$$V^{MTHFS} = V_{max}^{MTHFS} \frac{[5-THF]}{K_m^{MTHFS}+[5-THF]} \quad \text{(S2.9)}$$

SHMT (Serine hydroxymethyl transferase, EC 2.1.2.1). SHMT catalyzes reversible conversion between THF and serine and CH2-THF and glycine. Reaction mechanism is random sequential bi-bi [23]. The enzyme is inhibited by 5-THF and CH3-THF [24]. The inhibition constant value for 5-THF monoglutamate is equal to 130 µM [24]. Even after a 10 fold decrease that can be expected for polyglutamate this value remains high compared to 5-THF concentration in cells (Table 3). That is why we neglected this inhibition in the model. Inhibition of SHMT by CH3-THF is competitive with THF and CH2-THF [25]. The rate of SHMT reaction in the model is described by the following equation:

$$V^{SHMT} = \frac{\frac{V_{max}^{SHMT}}{K_{m,THF}K_{m,Ser}}\left([THF][Ser]-\frac{[CH2-THF][Gly]}{K_{eq}^{SHMT}}\right)}{1+\frac{[THF]}{K_{m,THF}^{SHMT}}+\frac{[Ser]}{K_{m,Ser}^{SHMT}}+\frac{[CH2-THF]}{K_{m,CH2-THF}^{SHMT}}+\frac{[Gly]}{K_{m,Gly}^{SHMT}}+\frac{[THF][Ser]}{K_{m,THF}^{SHMT}K_{m,Ser}^{SHMT}}+\frac{[CH2-THF][Gly]}{K_{m,CH2-THF}^{SHMT}K_{m,Gly}^{SHMT}}} \quad \text{(S2.10)}$$

where

$$K_{m,THF}^{SHMT} = K_{m0,THF}^{SHMT}\,(1+\frac{[CH3-THF]}{K_{i1,CH3-THF}^{SHMT}})$$

$$K_{m,CH2-THF}^{SHMT} = K_{m0,CH2-THF}^{SHMT}\,(1+\frac{[CH3-THF]}{K_{i2,CH3-THF}^{SHMT}})$$

TS (Thymidilate synthase, EC 2.1.1.45). TS catalyzes conversion of dUMP to dTMP via reductive methylation with CH2-THF. The reaction mechanism is ordered bi-bi with dUMP as the first substrate and DHF as the first product [26]. The enzyme is inhibited competitively by 10-THF [27]. According to the literature data the TS Michaelis constant value for dUMP lies in the range of 2.5 – 6.8 µM [26,28] that is several fold lower compared with physiological concentration of dUMP (Supplementary text S1). Thus, in cells the enzyme is saturated with dUMP and we used the Michaelis-Menten equation with one substrate including competitive inhibition with 10-THF to describe the TS reaction rate in the model:

$$V^{TS} = V_{max}^{TS}\frac{[CH2-THF]}{K_{m,CH2-THF}^{TS}\left(1+\frac{[10-THF]}{K_{i,10-THF}^{TS}}\right)+[CH_2-THF]} \quad \text{(S1.11)}$$

Synthesis of 5-THF. In cells 5-THF is produced from 10-THF or CH-THF in non-enzymatic reactions, as well as in side reactions catalyzed by MTHFC/D, SHMT, and, probably, by some

other enzymes [29,30]. In the model the rates of 5-THF production from 10-THF (V^{5THFS1}) and from CH-THF (V^{5THFS2}) are described by Michaelis-Menten equations with parameters presented in Supplementary text S4:

$$V^{5THFS1} = V_{max}^{5THFS1} \frac{[10-THF]}{K_m^{5THFS1}+[10-THF]} \quad \text{(S1.12)}$$

$$V^{5THFS2} = V_{max}^{5THFS2} \frac{[CH-THF]}{K_m^{5THFS2}+[CH-THF]} \quad \text{(S1.13)}$$

References

1. Mueller, W. T. & Benkovic, S. J. On the Purification and Mechanism of Action of 5-Aminoimidazole-4-carboxamide-Ribonucleotide Transformylase from Chicken Livery. *Biochemistry* **20,** 337–344 (1981).

2. Smith, S. L., Patrick, P., Stone, D., Phillips, A. W. & Burchall, J. J. Porcine liver dihydrofolate reductase. Purification, properties, and amino acid sequence. *J Biol Chem* **254,** 11475–11484 (1979).

3. Kutzbach, C. & Stokstad, E. L. R. 10-Formyl tetrahydrofolate: NADP oxidoreductase. *Methods Enzymol.* **18 part B,** 793–798 (1971).

4. Fox, J. T. & Stover, P. J. Folate-mediated one-carbon metabolism. in *Vitamins and hormones* **79,** 1–44 (2008).

5. Case, G. L., Kaisaki, P. J. & Steele, R. D. Resolution of rat liver 10-formyltetrahydrofolate dehydrogenase/hydrolase activities. *J Biol Chem* **263,** 10204–10207 (1988).

6. Cook, R. J. & Wagner, C. Enzymatic activities of rat liver cytosol 10-formyltetrahydrofolate dehydrogenase. *Arch Biochem Biophys* **321,** 336–344 (1995).

7. Kim, D. W., Huang, T., Schirch, D. & Schirch, V. Properties of tetrahydropteroylpentaglutamate bound to 10-formyltetrahydrofolate dehydrogenase. *Biochemistry* **35,** 15772–83 (1996).

8. Celeste, L. R. *et al.* Mechanism of N10-formyltetrahydrofolate synthetase derived from

complexes with intermediates and inhibitors. *Protein Sci.* **21,** 219–28 (2012).

9. McGuire, J. J. & Rabinowitz, J. C. Studies on the mechanism of formyltetrahydrofolate synthetase. The Peptococcus aerogenes enzyme. *J. Biol. Chem.* **253,** 1079–85 (1978).

10. Mejillano, M. R., Jahansouz, H., Matsunaga, T. O., Kenyon, G. L. & Himes, R. H. Formation and utilization of formyl phosphate by N10-formyltetrahydrofolate synthetase: evidence for formyl phosphate as an intermediate in the reaction. *Biochemistry* **28,** 5136–45 (1989).

11. Findlay, W. A., Zarkadas, C. G. & MacKenzie, R. E. An improved procedure for the purification of formiminotransferase-cyclodeaminase from pig liver. Kinetics of the transferase activity with tetrahydropteroylpolyglutamates. *Biochim. Biophys. Acta* **999,** 52–7 (1989).

12. Tabor, H. & Wyngarden, L. The enzymatic formation of formiminotetrahydrofolic acid, 5,10-methenyltetrahydrofolic acid, and 10-formyltetrahydrofolic acid in the metabolism of formiminoglutamic acid. *J Biol Chem* **234,** 1830–1846 (1959).

13. Uyeda, K. & Rabinowitz, J. C. Metabolism of Formiminoglycine. Formiminotetrahydrofolate cyclodeaminase. *J. Biol. Chem.* **242,** 24–31 (1967).

14. Paquin, J., Baugh, C. M. & MacKenzie, R. E. Channeling between the active sites of formiminotransferase-cyclodeaminase. Binding and kinetic studies. *J. Biol. Chem.* **260,** 14925–31 (1985).

15. Warren, L. & Buchanan, J. M. Biosynthesis of the purines. XIX. 2-Amino-N-ribosylacetamide 5'-phosphate (glycinamide ribotide) transformylase. *J. Biol. Chem.* **229,** 613–26 (1957).

16. Caperelli, C. A. Mammalian glycinamide ribonucleotide transformylase. Kinetic mechanism and associated de novo purine biosynthetic activities. *J Biol Chem* **264,** 5053–5057 (1989).

17. Banerjee, R. V, Frasca, V., Ballou, D. P. & Matthews, R. G. Participation of cob(I)alamin in the reaction catalyzed by methionine synthase from Escherichia coli: a steady-state and rapid reaction kinetic analysis. *Biochemistry* **29,** 11101–11109 (1990).

18. Chen, Z., Crippen, K., Gulati, S. & Banerjee, R. Purification and kinetic mechanism of a

mammalian methionine synthase from pig liver. *J.Biol.Chem.* **269,** 27193–27197 (1994).

19. Korendyaseva, T. K. *et al.* An allosteric mechanism for switching between parallel tracks in mammalian sulfur metabolism. *PLoS Comput Biol* **4,** e1000076 (2008).

20. Bertrand, R., MacKenzie, R. E. & Jolivet, J. Human liver methenyltetrahydrofolate synthetase: improved purification and increased affinity for folate polyglutamate substrates. *Biochim Biophys Acta* **911,** 154–161 (1987).

21. Huang, T. & Schirch, V. Mechanism for the coupling of ATP hydrolysis to the conversion of 5-formyltetrahydrofolate to 5,10-methenyltetrahydrofolate. *J Biol Chem* **270,** 22296–22300 (1995).

22. Hopkins, S. & Schirch, V. 5,10-Methenyltetrahydrofolate synthetase. Purification and properties of the enzyme from rabbit liver. *J Biol Chem* **259,** 5618–5622 (1984).

23. Schirch, L. V *et al.* Serine transhydroxymethylase: evidence for a sequential random mechanism. *Biochemistry* **16,** 410–419 (1977).

24. Schirch, L. & Ropp, M. Serine transhydroxymethylase. Affinity of tetrahydrofolate compounds for the enzyme and enzyme-glycine complex. *Biochemistry* **6,** 253–7 (1967).

25. Matthews, R. G., Ross, J., Baugh, C. M., Cook, J. D. & Davis, L. Interactions of pig liver serine hydroxymethyltransferase with methyltetrahydropteroylpolyglutamate inhibitors and with tetrahydropteroylpolyglutamate substrates. *Biochemistry* **21,** 1230–1238 (1982).

26. Lu, Y. Z., Aiello, P. D. & Matthews, R. G. Studies on the polyglutamate specificity of thymidylate synthase from fetal pig liver. *Biochemistry* **23,** 6870–6876 (1984).

27. Balinska, M., Rhee, M., Whiteley, J. M., Priest, D. G. & Galivan, J. Inhibition of mammalian thymidylate synthase by 10-formyltetrahydropteroylpolyglutamate. *Arch. Biochem. Biophys.* **284,** 219–222 (1991).

28. Nakata, R., Tsukamoto, I., Miyoshi, M. & Kojo, S. Purification and characterization of thymidylate synthetase from rat regenerating liver. *Biochim Biophys Acta* **924,** 297–302 (1987).

29. Baggott, J. E. & MacKenzie, R. E. 5,10-methenyltetrahydrofolate cyclohydrolase, rat liver and chemically catalysed formation of 5-formyltetrahydrofolate. *Biochem J* **374,** 773–778

(2003).

30. Stover, P. & Schirch, V. Synthesis of (6S)-5-formyltetrahydropteroyl-polyglutamates and interconversion to other reduced pteroylpolyglutamate derivatives. *Anal Biochem* **202,** 82–88 (1992).

Supplementary text S3. Methylenetetrahydrofolate dehydrogenase/Methylenetetrahydrofolate cyclohydrolase (EC1.5.1.5 / EC 3.5.4.9)

Methylenetetrahydrofolate dehydrogenase and methylenetetrahydrofolate cyclohydrolase reactions are catalyzed by the same cytoplasmic protein that also possesses formyltetrahydrofolate synthetase activity. Both, methylenetetrahydrofolate dehydrogenase and methylenetetrahydrofolate cyclohydrolase activities associate with one (N-terminal) domain of the enzyme while formyltetrahydrofolate synthase activity associates with another (C-terminal) domain [1]. That is why we consider the formyltetrahydrofolate synthetase reaction as an independent activity.

Methylenetetrahydrofolate dehydrogenase activity (MTHFD - EC 1.5.1.5) provides the reversible NADP/NADPH dependent transformation between CH2-THF and CH-THF. Methylenetetrahydrofolate cyclohydrolase activity (MTHFC – EC 3.5.4.9) provides reversible transformation between 10-THF and CH-THF. Interestingly, the methylenetetrahydrofolate cyclohydrolase activity is detected mostly in the absence of NADP/NADPH cofactors in vitro [2,3]. In the presence of the cofactors (under physiological conditions) almost all consumed 10-THF is converted to CH2-THF without releasing CH-THF that demonstrates channeling between MTHFC and MTHFD. Also, in MTHFD reaction only 50% of CH2-THF is converted to CH-THF while the rest is channeled to 10-THF [4].

Based on the literature data [5] we suggested the scheme of reactions provided by MTHFD and MTHFC which is shown in Fig. S3.1. We assumed random sequential binding of all folates and cofactors to the enzyme in both MTHFD and channeling (MTHFD/C) reactions. According to the scheme the enzyme can be involved only in one reaction: MTHFD, MTHFC, or MTHFD/C. In other words, the reactions cannot be catalyzed independently. MTHFC reaction can proceed only between corresponding enzyme-folate complexes lacking bound NADP/NADPH cofactors. The channeling between 10-THF and CH2-THF in the scheme is provided due to the absence of direct conversion of E•NADPH•10-THF complex to E•NADPH•CH-THF complex and vise-versa (Fig. S3.1). To provide 50% channeling of CH2-THF to 10-THF in the scheme we set the rate constant of forward MTHFD reaction (k_{Df}) equal to the rate constant of forward channeling reaction (k_{Cf}) (Fig. S3.1).

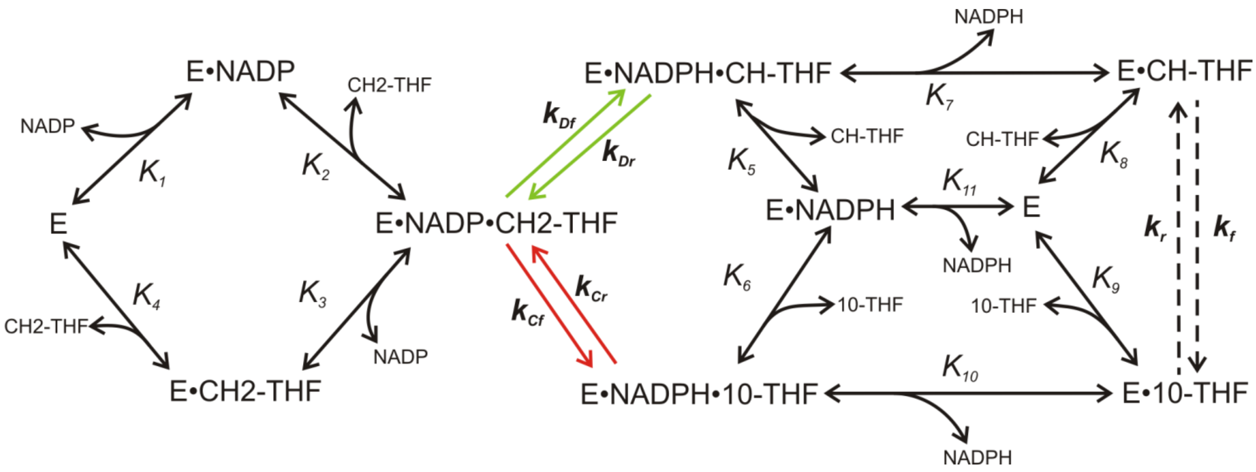

Fig. S3.1. Scheme of reactions catalyzed by methylenetetrahydrofolate dehydrogenase and methylenetetrahydrofolate cyclohydrolase. E denotes enzyme. Solid black arrows show association-dissociation of corresponding enzyme-substrate complexes. K_1-K_{11} denote corresponding dissociation constants. Black dashed arrows show forward and reverse MTHFC reactions with forward (k_f) and reverse (k_r) rate constants. Green and red arrows show MTHFD and channeling reactions respectively with forward (k_{Df} and k_{Cf}) and reverse (k_{Dr} and k_{Cr}) rate constants.

Using the scheme in Fig. S3.1, one can obtain equations for the reaction rates.
To make the equations less bulky let us introduce the following designations:

CH2-THF → CH2
CH-THF → CH
10-THF → CHO
NADPH→NPH
NADP→NP

V^{MTHFC} - the rate of methyltetrahydrofolate cyclohydrolase reaction without NADPH
V^{MTHFD} - the rate of methyl tetrahydrofolate dehydrogenase reaction
$V^{MTHFD/C}$ - the channeling rate between CH_2-THF and 10-THF

The positive direction of the V^{MTHFC}, V^{MTHFD}, and $V^{MTHFD/C}$ rates correspond to the reaction rate constants with index *f* in Fig. S3.1.
The rates of MTHFC, MTHFD, and MTHFC/D reactions are described by the following equations:

$$V^{MTHFC} = [E.CH] * k_f - [E.CHO] * k_r$$
$$V^{MTHFD} = [E.NP.CH2] * k_{Df} - [E.NPH.CH] * k_{Dr} \quad \text{(S3.1)}$$
$$V^{MTHFD/C} = [E.NP.CH2] * k_{Cf} - [E.NPH.CHO] * k_{Cr}$$

Here expressions in square brackets denote concentrations of corresponding enzyme-substrate complexes. Assuming a rapid equilibrium in all stages of the scheme in Fig. S3.1 except the catalytic stages, we obtain the following system of equations:

$$K_1 = \frac{[NP]*[E]}{[E.NP]}$$

$$K_2 = \frac{[E.NP]*[CH2]}{[E.NP.CH2]}$$

$$K_4 = \frac{[E]*[CH2]}{[E.CH2\,]}$$

$$K_5 = \frac{[E.NPH]*[CH]}{[E.NPH.CH]}$$

$$K_6 = \frac{[E.NPH]*[CHO]}{[E.NPH.CHO]} \quad \text{(S3.2)}$$

$$K_8 = \frac{[E]*[CH]}{[E.CH]}$$

$$K_9 = \frac{[E]*[CHO]}{[E.CHO]}$$

$$K_{11} = \frac{[E]*[NPH]}{[E.NPH]}$$

$$[\mathrm{E_0}] = [E] + [E.NP] + [E.NPH] + [E.NP.CH2] + [E.NPH.CH] + [E.NPH.CHO] + [E.CH] + [E.CHO] + [E.CH2]$$

Using the system of equations (S3.2), we obtain equations for the concentrations of enzyme-substrate complexes:

$$[E.NP.CH2] = \frac{[E_0][NP][CH2]}{[NP][CH2]+K_1K_2\left(1+\frac{[NP]}{K_1}+\frac{[NPH]}{K_{11}}\left(1+\frac{[CH]}{K_5}+\frac{[CHO]}{K_6}\right)+\frac{[CHO]}{K_9}+\frac{[CH]}{K_8}+\frac{[CH2]}{K_4}\right)}$$

$$[E.NPH.CH] = \frac{[E_0][NPH][CH]}{[NPH][CH]+K_5K_{11}\left(1+\frac{[NP]}{K_1}\left(1+\frac{[CH2]}{K_2}\right)+\frac{[NPH]}{K_{11}}\left(1+\frac{[CHO]}{K_6}\right)+\frac{[CHO]}{K_9}+\frac{[CH]}{K_8}+\frac{[CH2]}{K_4}\right)}$$

$$[E.CH] = \frac{[E_0][CH]}{[CH]+K_8\left(1+\frac{[NP]}{K_1}\left(1+\frac{[CH2]}{K_2}\right)+\frac{[NPH]}{K_{11}}\left(1+\frac{[CH]}{K_5}+\frac{[CHO]}{K_6}\right)+\frac{[CHO]}{K_9}+\frac{[CH2]}{K_4}\right)} \quad \text{(S3.3)}$$

$$[E.CHO] = \frac{[E_0][CHO]}{[CHO]+K_9\left(1+\frac{[NP]}{K_1}\left(1+\frac{[CH2]}{K_2}\right)+\frac{[NPH]}{K_{11}}\left(1+\frac{[CH]}{K_5}+\frac{[CHO]}{K_6}\right)+\frac{[CH]}{K_8}+\frac{[CH2]}{K_4}\right)}$$

$$[E.NPH.CHO] = \frac{[E_0][NPH][CHO]}{[NPH][CHO]+K_6K_{11}\left(1+\frac{[NP]}{K_1}\left(1+\frac{[CH2]}{K_2}\right)+\frac{[NPH]}{K_{11}}\left(1+\frac{[CH]}{K_5}\right)+\frac{[CHO]}{K_9}+\frac{[CH]}{K_8}+\frac{[CH2]}{K_4}\right)}$$

And finally, using equations (S3.3) and (S3.1) we obtain the equations for the rates of MTHFC, MTHFD and MTHFD/C reactions:

$$V^{MTHFC} = \frac{[E_0][CH]k_f}{[CH]+K_8\left(1+\frac{[NP]}{K_1}\left(1+\frac{[CH2]}{K_2}\right)+\frac{[NPH]}{K_{11}}\left(1+\frac{[CH]}{K_5}+\frac{[CHO]}{K_6}\right)+\frac{[CHO]}{K_9}+\frac{[CH2]}{K_4}\right)} - \frac{[E_0][CHO]k_r}{[CHO]+K_9\left(1+\frac{[NP]}{K_1}\left(1+\frac{[CH2]}{K_2}\right)+\frac{[NPH]}{K_{11}}\left(1+\frac{[CH]}{K_5}+\frac{[CHO]}{K_6}\right)+\frac{[CH]}{K_8}+\frac{[CH2]}{K_4}\right)} \quad \text{(S3.4)}$$

$$V^{MTHFD} = \frac{[E_0][NP][CH2]k_{Df}}{[NP][CH2]+K_1K_2\left(1+\frac{[NP]}{K_1}+\frac{[NPH]}{K_{11}}\left(1+\frac{[CH]}{K_5}+\frac{[CHO]}{K_6}\right)+\frac{[CHO]}{K_9}+\frac{[CH]}{K_8}+\frac{[CH2]}{K_4}\right)} - \\ - \frac{[E_0][NPH][CH]k_{Dr}}{[NPH][CH]+K_5K_{11}\left(1+\frac{[NP]}{K_1}\left(1+\frac{[CH2]}{K_2}\right)+\frac{[NPH]}{K_{11}}\left(1+\frac{[CHO]}{K_6}\right)+\frac{[CHO]}{K_9}+\frac{[CH]}{K_8}+\frac{[CH2]}{K_4}\right)} \quad (S3.5)$$

$$V^{MTHFD/C} = \frac{[E_0][NP][CH2]k_{Cf}}{[NP][CH2]+K_1K_2\left(1+\frac{[NP]}{K_1}+\frac{[NPH]}{K_{11}}\left(1+\frac{[CH]}{K_5}+\frac{[CHO]}{K_6}\right)+\frac{[CHO]}{K_9}+\frac{[CH]}{K_8}+\frac{[CH2]}{K_4}\right)} - \\ - \frac{[E_0][NPH][CHO]k_{Cr}}{[NPH][CHO]+K_6K_{11}\left(1+\frac{[NP]}{K_1}\left(1+\frac{[CH2]}{K_2}\right)+\frac{[NPH]}{K_{11}}\left(1+\frac{[CH]}{K_5}\right)+\frac{[CHO]}{K_9}+\frac{[CH]}{K_8}+\frac{[CH2]}{K_4}\right)} \quad (S3.6)$$

The equation parameter values were taken from the literature or calculated from the literature data. We choose parameter values consistent with Haldane relationships for the enzymatic reactions.

$$K_{eq1} = \frac{[CHO][NPH]}{[CH2\ ][NP]} = \frac{K_{Cf}K_6K_{11}}{K_{Cr}K_1K_2} \quad \text{(S3.7)}$$

$$K_{eq2} = \frac{[CHO]}{[CH]} = \frac{K_fK_9}{K_rK_8} \quad \text{(S3.8)}$$

$$K_{eq3} = \frac{[CH][NPH]}{[CH2][NP]} = \frac{K_{Df}K_5K_{11}}{K_{Dr}K_1K_2} \quad \text{(S3.9)}$$

Here K_{eq1}, K_{eq2}, *and* K_{eq3} denote equilibrium constants for MTHFD/C, MTHFC, and MTHFD reactions respectively.

The equation parameter values and corresponding comments are presented in Table S3.1.

Table S3.1. **Kinetic parameters of MTHFD and MTHFC (EC 1.5.1.5/EC 3.5.4.9).**

Values for all catalytic constants obtained at temperature other than 37 ^{o}C were recalculated to values at 37 ^{o}C using temperature dependence coefficient of 2 per 10 ^{o}C. In several cases literature data for Michaelis constant values were used to evaluate values of corresponding dissociation constants. Similar to all other enzyme kinetic parameters (Supplementary text S4) for MTHFD/C we used Michaelis, inhibition, and dissociation constant values for folates obtained with polyglutamate forms of folates, if available. If data obtained with polyglutamate forms of folates were not available we used data obtained with monoglutamates decreased by factor of about ten. The exact parameters values in these cases were chosen to satisfy the Haldane equations (S3.7-S3.9) and detailed balance equations.

Parameter (Units)	Model	Experiment	Comments and references
E_0 (µM)	3.3	3.3	Pig liver [6].
k_{Df} (1/s)	48	18	Recombinant human liver cytoplasmic enzyme [7].
		50	Pig liver [2].
		38[a]	Rat liver, calculated from [6,8].
k_{Dr} (1/s)	63	60	Recombinant human liver cytoplasmic enzyme [7].
k_f (1/s)	189	250	Recombinant human liver cytoplasmic enzyme [7].
		320	Pig liver [2].
		134[b]	Rat liver, calculated from [6,8].

k_r (1/s)	24	25	Recombinant human liver cytoplasmic enzyme [7].
k_{Cf} (1/s)	48	-	$k_{Cf} = k_{Df}$ according to 50% channeling in forward MTHFD reaction [4]
k_{Cr} (1/s)	24	25	Recombinant human liver cytoplasmic enzyme [7].
K_1 (µM)	9.6	-	Calculated from the detailed balance equation: $K_1 \cdot K_2 = K_3 \cdot K_4$.
K_2 (µM)	3	3.0	Pig liver, *Km* for CH2-THF pentaglutamate for MTHFD [9].
		2.4 - 4.2	Pig liver, *Km* for CH2-THF pentaglutamate for MTHFD [10].
K_3 (µM)	8	9-13	Pig liver, *Km* for NADP for MTHFD with CH2-THF pentaglutamate [10].
K_4 (µM)	3.6	39	Recombinant human liver cytoplasmic enzyme, *Kd* for CH2-THF monoglutamate in the absence of NADP/NADPH [5].
K_5 (µM)	0.1	-	Calculated from equation (S3.9).
K_6 (µM)	0.8	7	Recombinant human liver cytoplasmic enzyme, *Kd* for 10-THF monoglutamate in the presence of NADP/NADPH [5].
K_8 (µM)	6	55	Mouse liver, *Km* for CH-THF monoglutamate for MTHFC [11].
		43	Recombinant human liver cytoplasmic enzyme, *Km* for CH-THF monoglutamate for MTHFC [7].
		16.4	Recombinant human liver cytoplasmic enzyme, *Km* for CH-THF monoglutamate for MTHFC [12].
K_9 (µM)	16	190	Recombinant human liver cytoplasmic enzyme, *Kd* for 10-THF monoglutamate for MTHFC [5].

		51	Recombinant human liver cytoplasmic enzyme, *Km* for 10-THF monoglutamate for MTHFC [7].
K_{10} (µM)	14.4	13	Recombinant human liver cytoplasmic enzyme, *Km* for NADPH for overall reverse MTHFD/C reaction with 10-THF monoglutamate [7].
K_{11} (µM)	288	-	Calculated from the detailed balance equation: $K_{11}{\cdot}K_6 = K_9{\cdot}K_{10}$
K_{eq1}	16	16	Recombinant human liver cytoplasmic enzyme [5]
K_{eq2}	21	21	Calculated from the nonenzymatic first order reaction rate constants [7]
K_{eq3}	0.76	0.76[c]	Calculated from [5,7]

[a] Using MTHFD activity in rat liver V_{max}^{MTHFD} = 456 mmol/h kg tissue [8] and liver enzyme concentration of 3.3 µM [6], one can get the value for k_{Df} = 456/(3.3*3.6) ≈ 38 1/s.

[b] Using MTHFC activity in rat liver V_{max}^{MTHFC} = 1596 mmol/h kg tissue [8] and liver enzyme concentration of 3.3 µM [6], one can get the value for k_f = 1596/(3.3*3.6) ≈ 134 1/s.

[c] Taking values for K_{eq1} = 16 [5] and for K_{eq2} = 21 [7] and using equations S3.7-S3.9 one can get the value for $K_{eq3} = K_{eq1}/K_{eq2}$ = 0.76.

References

1. Howard, K. M., Muga, S. J., Zhang, L., Thigpen, A. E. & Appling, D. R. Characterization of the rat cytoplasmic C1-tetrahydrofolate synthase gene and analysis of its expression in liver regeneration and fetal development. *Gene* **319,** 85–97 (2003).

2. Tan, L. U., Drury, E. J. & MacKenzie, R. E. Methylenetetrahydrofolate dehydrogenase-methenyltetrahydrofolate cyclohydrolase-formyltetrahydrofolate synthetase. A multifunctional protein from porcine liver. *J Biol Chem* **252,** 1117–1122 (1977).

3. Schirch, L. V. Formyl-Methenyl-Methylenetetrahydrofolate Synthetase from Rabbit Liver (Combined). Evidence for a Single Site in the Conversion of 5,10-Methylenetetrahydrofolate to 10-Formyltetrahydrofolate. *Arch Biochem Biophys* **189,** 283–290 (1978).

4. Pawelek, P. D., Allaire, M., Cygler, M. & MacKenzie, R. E. Channeling efficiency in the bifunctional methylenetetrahydrofolate dehydrogenase/cyclohydrolase domain: The effects of site-directed mutagenesis of NADP binding residues. *Biochim. Biophys. Acta - Protein Struct. Mol. Enzymol.* **1479,** 59–68 (2000).

5. Pelletier, J. N. & MacKenzie, R. E. Binding and interconversion of tetrahydrofolates at a single site in the bifunctional methylenetetrahydrofolate dehydrogenase/cyclohydrolase. *Biochemistry* **34,** 12673–12680 (1995).

6. Green, J. M., MacKenzie, R. E. & Matthews, R. G. Substrate flux through methylenetetrahydrofolate dehydrogenase: predicted effects of the concentration of methylenetetrahydrofolate on its partitioning into pathways leading to nucleotide biosynthesis or methionine regeneration. *Biochemistry* **27,** 8014–8022 (1988).

7. Pawelek, P. D. & MacKenzie, R. E. Methenyltetrahydrofolate cyclohydrolase is rate limiting for the enzymatic conversion of 10-formyltetrahydrofolate to 5,10-methylenetetrahydrofolate in bifunctional dehydrogenase-cyclohydrolase enzymes. *Biochemistry* **37,** 1109–1115 (1998).

8. Cheek, D. W., Appling, D. R., Cheek, W. D. & Appling, D. R. Purification, Immunoassay, and Tissue Distribution of Rat C1-Tetrahydrofolate Synthase. *Arch. Biochem. Biophys.* **270,** 504–512 (1989).

9. Mackenzie, R. E. & Baugh, C. M. Tetrahydropterolypolyglutamate derivatives as substrates of two multifunctional proteins with folate-dependent enzyme activities. *Biochim. Biophys. Acta* **611,** 187–195 (1980).

10. Ross, J., Green, J., Baugh, C. M., MacKenzie, R. E. & Matthews, R. G. Studies on the polyglutamate specificity of methylenetetrahydrofolate dehydrogenase from pig liver. *Biochemistry* **23,** 1796–1801 (1984).

11. Gardam, M. A., Mejia, N. R. & MacKenzie, R. E. The NADP-dependent trifunctional methylenetetrahydrofolate dehydrogenase purified from mouse liver is immunologically distinct from the mouse NAD-dependent bifunctional enzyme. *Biochem. Cell Biol.* **66,** 66–70 (1988).

12. Pelletier, J. N. & MacKenzie, R. E. Binding to the 2’,5’-ADP Subsite Stimulates Cyclohydrolase Activity of Human NADP+-Dependent Methylenetetrahydrofolate Dehydrogenase/Cyclohydrolase. *Biochemistry* **33,** 1900–1906 (1994).

Supplementary text S4. Enzyme kinetic parameters.

In the model we tried to used enzyme kinetic parameters obtained for rodent liver, if available, making preference for rat liver. All enzyme activities used in the model were obtained for rat liver and presented as mmol per hour per kg of wet liver, that is a very close estimation for mmol per hour per liter. In case if enzyme activity in the literature was normalized to protein we recalculated the activity as per kg using protein concentration of 200 g per kg of wet liver [1]. If enzyme activity or catalytic constant in the literature was measured at temperature other than 37 ^{o}C, its value was recalculated for 37 ^{o}C using temperature coefficient of 2 per 10 ^{o}C. In cells folates exist mainly in the polyglutamate forms containing 5-6 glutamate residues per folate molecule. Such polyglutamate forms have 2 - 100 times higher affinity to enzymes compared with monoglutamate forms [2–4]. While folate Michaelis, inhibition, and dissociation constant values obtained at different conditions are presented in Table S4.1, only those, obtained for the polyglutamate forms of folates were used in the model. In a few cases when constant for polyglutamate was not available in the literature we used value of constant obtained for monoglutamate divided by ten. Commetnts in the Table S4.1 describe enzyme source and, glutamination status of folates. If nothing is mentioned about glutamination satatus it means that data were obtained with monoglutamate form of the corresponding folate.

Table S4.1. Enzyme kinetic parameters.

Parameter (Units)	Model	Experiment	Comments and References
**AT (AICAR transformylase, EC 2.1.2.3)**			
V_{max}^{AT} (mmol/h kg liver)	8.8	8.8	Rat liver [5].
$K_{m,10-THF}^{AT}$ (µM)	5.9	5.9	Human breast cancer cells MCF-7, 10-THF pentaglutamate [6].
$K_{m,AICAR}^{AT}$ (µM)	10	10	Human recombinant enzyme [7].
**DHFR (Dihydrofolate reductase, EC 1.5.1.3)**			
V_{max}^{DHFR}	4.9	109	Rat liver [8].
		57	Rat liver [9].

(mmol/h kg liver)		4.9	Rat liver [10].
$K_{m,DHF}^{DHFR}$ (μM)	0.034	0.1	Rat liver [11].
		0.74	Pig liver [12].
		0.17	Rat liver [13].
$K_{m,NADPH}^{DHFR}$ (μM)	The enzyme is saturated with NADPH	3.2	Pig liver [12].
		0.72	Rat liver [13].
		0.2	Rabbit liver [14].
**FTHFD (Formyltetrahydrofolate dehydrogenase, EC 1.5.1.6).**			
V_{max}^{FTHFD} (mmol/h kg liver)	25	45	Rat liver [15].
		14	Rat liver [16].
		29	Rat liver [17].
		11.2	Rat liver [18].
		23.1	Rat liver [19].
		26.1	Rat liver [20].
$K_{m,NADP}^{FTHFD}$ (μM)	The enzyme is saturated with NADP	0.9	Rat liver [17].
		1	Rat liver [21].
		3.5	Pig liver [22].
$K_{i,THF}^{FTHFD}$ (μM)	0.015	0.015	Rabbit liver, THF pentaglutamate [23].
		1	Pig liver [22].
$K_{m,10-THF}^{FTHFD}$ (μM)	0.9	0.9	Rabbit liver, 10-THF pentaglutamate [23].
**FTHFS (Formyltetrahydrofolate synthetase, EC 6.3.4.3)**			
V_{max}^{FTHFS} (mmol/h kg liver)	432	162	Rat liver [9].
		384	Rat liver [24].
		504	Rat liver [25].
$K_{eq}^{FTHFS} = \frac{[ADP][P_i][10-THF]}{[ATP][HCOOH][THF]}$	53	115	[26]
		20-60	[27]

$K_{m,THF}^{FTHFS}$ (μM)	1	239	Mouse liver [28].
		15	Rabbit liver [29].
		<1	Rabbit liver, THF pentaglutamate. [30].
$K_{m,ATP}^{FTHFS}$ (μM)	The enzyme is saturated with ATP	47	Mouse liver [28].
		67	Rabbit liver [29].
		40	Rabbit liver, with THF pentaglutamate [30].
$K_{m,HCOOH}^{FTHFS}$ (μM)	4	18700	Mouse liver [28].
		166	Rabbit liver [29].
		4	Rabbit liver, with THF pentaglutamate [30].
$K_{m,ADP}^{FTHFS}$ (μM)	The enzyme is saturated with ADP	130	C. cylindrosporum [31]
$K_{m,H2PO4}^{FTHFS}$ (μM)	5000	5000	C. cylindrosporum [32].
$K_{m,10-THF}^{FTHFS}$ (μM)	120	10000	C. cylindrosporum [31].
		120	C. cylindrosporum, 10-THF triglutamate [31].
**GFIT/FITCD (Glutamate formimidoyltransferase/formimidoyltetrahydrofolate cyclodeaminase, EC 2.1.2.5/EC 4.3.1.4)**			
$V_{max}^{GFIT/FITCD}$ (mmol/h kg liver)	216	215	Rat liver [19].
		213	Rat liver [20].
$K_{m,THF}^{GFIT/FITCD}$ (μM)	2	3.4	Pig liver, THF pentaglutamate.[33].
		0.7	Pig liver, THF pentaglutamate [34].
		100	Pig liver [35].
$K_{m,FIGlu}^{GFIT/FITCD}$ (μM)	400	12000	Pig liver [36].
		11000	Pig liver [35].
		5800	Pig liver [37]
		400	Pig liver, with THF pentaglutamate [38].
**GT (GAR transformylase, EC 2.1.2.2)**			
V_{max}^{GT} (mmol/h kg liver)	0.8	1.6	Rat liver [5].
		0.7	Rat liver [39].

$K_{m,10-THF}^{GT}$ (µM)	0.8	15	Mouse leukemia cells L1210 [39].
		0.8	Mouse lymphoma cells L5178Y [40].
$K_{m,GAR}^{GT}$ (µM)	10	17	Mouse leukemia cells L1210 [39].
		2.5	Mouse lymphoma cells L5178Y [41].
$K_{i,10-THF}^{GT}$ (µM)	0.36	3.6	Mouse lymphoma cells L5178Y [41].
**MS (Methionine synthase, EC 2.1.1.13)**			
V_{max}^{MS} (mmol/h kg liver)	0.59	3.1	Rat liver [42].
		0.5	Rat liver [18].
		1.2	Rat liver [24].
		0.9	Rat liver [43].
$K_{m,CH3-THF}^{MS}$ (µM)	4	4	Rat liver, CH3-THF pentaglutamate [44].
		89	Recombinant rat enzyme [45].
		12.8	Pig liver [46].
		0.5-8.4	Pig liver, THF hexaglutamate [47].
$K_{m,Hcy}^{MS}$ (µM)	2.5	2.6	Recombinant rat enzyme [45].
		2.2	Pig liver [46].
$K_{d,CH3-THF}^{MS}$ (µM)	14.2	142	Pig liver [46].
**MTHFD/MTHFC (Methylenetetrahydrofolate dehydrogenase/methylenetetrahydrofolate cyclohydrolase (EC1.5.1.5 / EC 3.5.4.9)** Reaction rate equations and kinetic parameters for MTHFD and MTHFC are presented in Supplementary text S3.			
**MTHFR (Methylenetetrahydrofolate reductase, EC 1.5.1.20)**			
V_{max}^{MTHFR} (mmol/h kg liver)	0.9	3.4	Rat liver [48].
		0.7	Rat liver [18].
		0.34	Rat liver [19].
		1.9	Rat liver [49].
$K_{m,CH2-THF}^{MTHFR}$ (µM)	2	88	Pig liver, [50].

		19	Pig liver, [51].
		0.1-1.7	Pig liver, CH2-THF from triglutamates to hexaglutamates [52].
$K_{m,NADPH}^{MTHFR}$ (µM)	120	16	Pig liver [51].
		15-185	Pig liver, with CH2-THF from triglutamates to hexaglutamates [52].
$K_{i,CH3-THF}^{MTHFR}$ (µM)	2	20	Pig liver [53].
$K_{i,AdoMet}^{MTHFR}$ (µM)	3	0.9	Pig liver [54].
		3	Pig liver [55].
$K_{i,AdoHcy}^{MTHFR}$ (µM)	3	3	Pig liver [56].
**MTHFS (Methenyltetrahydrofolate synthase, EC 6.3.3.2)**			
V_{max}^{MTHFS} (mmol/h kg liver)	12.6	14	Rat liver [57].
		8	Human liver [58].
$K_{m,5-THF}^{MTHFS}$ (µM)	0.5	0.6	Human liver, 5-THF-pentaglutamate [59].
		0.5	Rabbit liver [60].
		5	Recombinant mouse enzyme [61].
		8	Rabbit liver [62].
		0.2	Rabbit liver, 5-THF-pentaglutamate [14].
$K_{m,ATP}^{MTHFS}$ (µM)	The enzyme is saturated with ATP	300	Rabbit liver [60].
		20	Human liver [59]
		300	Rabbit liver [14].
		769	Recombinant mouse enzyme [61].
**SHMT (Serine hydroxymethyl transferase, EC 2.1.2.1)**			
V_{max}^{SHMT} (mmol/h kg liver)	500	400	Rat liver [9].
		758	Rat liver [8].
		216	Rat liver [63].

$K_{eq}^{SHMT} = \frac{[CH2-THF][Gly]}{[THF][Ser]}$	8	8 - 12	[64]
$K_{m,Ser}^{SHMT}$ (µM)	1000	510	Rat liver [65].
		210	Pig liver, with THF-triglutamate [66].
		1000	Rabbit liver, with THF hexaglutamate [3].
		700	Monkey liver [67].
		360	Rabbit liver [64].
$K_{m,Gly}^{SHMT}$ (µM)	5000	5000	Rabbit liver, with CH2-THF hexaglutamate [3].
		1700	Rabbit liver [64].
$K_{m0,THF}^{SHMT}$ (µM)	0.5	1.7	Pig liver, THF-triglutamate [66].
		0.1-0.5	Rabbit liver, THF-hexaglutamate [3].
		4.1	Human recombinant enzyme [68].
		10	Rabbit recombinant enzyme [68].
		40-60	Rabbit liver [69].
$K_{m0,CH2-THF}^{SHMT}$ (µM)	1.7	17	Rabbit liver [64].
$K_{i1,CH3-THF}^{SHMT}$ (µM)	3	30	Pig liver. Calculated for monoglutamate using data from [64].[b]
$K_{i2,CH3-THF}^{SHMT}$ (µM)	14	140	Pig liver. Calculated for monoglutamate using data from [64].[b]
**TS (Thymidylate synthase EC 2.1.1.45)**			
V_{max}^{TS} (mmol/h kg liver)	0.09	0.04	Rat liver [70].
		0.12	Rat liver [71].
		0.14	Rat liver [1].
$K_{m,dUMP}^{TS}$ (µM)	The enzyme is saturated with dUMP	6.8	Rat liver [72].
		2.5	Rat liver [73].
$K_{m,CH2-THF}^{TS}$ (µM)	0.7	65	Rat liver [72].
		2	Rat liver [73].
		0.7	Rat hepatoma cells H35, CH2-THF

			heptaglutamate [74].
$K_{i,10-THF}^{TS}$ (μM)	0.1	0.1	Rat hepatoma cells H35, 10-THF heptaglutamate [74].
**Synthesis of 5-THF**			
V_{max}^{5THFS1} (mmol/h/l liver)	0.015	0.015	Rat liver [57].
$K_{m,10-THF}^{5THFS1}$ (μM)	0.9	9	Rat liver [57].
V_{max}^{5THFS2} (mmol/h/l liver)	6.5	6.5	Rabbit liver, calculated from [3,75].
$K_{m,CH-THF}^{5THFS2}$ (μM)	4	40	Rabbit liver [75].

References

1. Komatsu, M. & Tsukamoto, I. Effect of folic acid on thymidylate synthase and thymidine kinase in regenerating rat liver after partial hepatectomy. *Biochim. Biophys. Acta* **1379,** 289–296 (1998).

2. Green, J. M., MacKenzie, R. E. & Matthews, R. G. Substrate flux through methylenetetrahydrofolate dehydrogenase: predicted effects of the concentration of methylenetetrahydrofolate on its partitioning into pathways leading to nucleotide biosynthesis or methionine regeneration. *Biochemistry* **27,** 8014–8022 (1988).

3. Strong, W. B. & Schirch, V. In vitro conversion of formate to serine: effect of tetrahydropteroylpolyglutamates and serine hydroxymethyltransferase on the rate of 10-formyltetrahydrofolate synthetase. *Biochemistry* **28,** 9430–9439 (1989).

4. Shane, B. Folylpolyglutamate synthesis and role in the regulation of one-carbon metabolism. *Vitam. Horm.* **45,** 263–335 (1989).

5. Deacon, R., Perry, J., Lumb, M. & Chanarin, I. Effect of cobalamin inactivation on folate-dependent transformylases involved in purine synthesis in rats. *Biochem J* **227,** 67–71 (1985).

6. Baram, J. *et al.* Identification and biochemical properties of 10-formyldihydrofolate, a

novel folate found in methotrexate-treated cells. *J. Biol. Chem.* **263,** 7105–7111 (1988).

7. Bulock, K. G., Beardsley, G. P. & Anderson, K. S. The kinetic mechanism of the human bifunctional enzyme ATIC (5-amino-4-imidazolecarboxamide ribonucleotide transformylase/inosine 5'-monophosphate cyclohydrolase). A surprising lack of substrate channeling. *J Biol Chem* **277,** 22168–22174 (2002).

8. Rovinetti, C., Bovina, C., Tolomelli, B. & Marchetti, M. Effects of testosterone on the metabolism of folate coenzymes in the rat. *Biochem J* **126,** 291–294 (1972).

9. Barbiroli, B., Bovina, C., Tolomelli, B. & Marchetti, M. Folate metabolism in the rat liver during regeneration after partial hepatectomy. *Biochem J* **152,** 229–232 (1975).

10. Sica, L., Gilli, R., Briand, C. & Sari, J. C. A flow microcalorimetric method for enzyme activity measurements: application to dihydrofolate reductase. *Anal Biochem* **165,** 341–348 (1987).

11. Stone, K. J. The role of tetrahydrofolate dehydrogenase in the hepatic supply of tetrahydrobiopterin in rats. *Biochem J* **157,** 105–109 (1976).

12. Smith, S. L., Patrick, P., Stone, D., Phillips, A. W. & Burchall, J. J. Porcine liver dihydrofolate reductase. Purification, properties, and amino acid sequence. *J Biol Chem* **254,** 11475–11484 (1979).

13. Webber, S. & Whiteley, J. M. Comparative activity of rat liver dihydrofolate reductase with 7,8-dihydrofolate and other 7,8-dihydropteridines. *Arch Biochem Biophys* **236,** 681–690 (1985).

14. Schirch, V. Purification of folate-dependent enzymes from rabbit liver. *Methods Enzymol.* **281,** 146–161 (1997).

15. Case, G. L., Kaisaki, P. J. & Steele, R. D. Resolution of rat liver 10-formyltetrahydrofolate dehydrogenase/hydrolase activities. *J Biol Chem* **263,** 10204–10207 (1988).

16. Min, H., Im, E. S., Seo, J. S., Mun, J. A. & Burri, B. J. Effects of chronic ethanol ingestion and folate deficiency on the activity of 10-formyltetrahydrofolate dehydrogenase in rat liver. *Alcohol Clin Exp Res* **29,** 2188–2193 (2005).

17. Scrutton, M. C. & Beis, I. Inhibitory effects of histidine and their reversal. The roles of pyruvate carboxylase and N10-formyltetrahydrofolate dehydrogenase. *Biochem J* **177,** 833–846 (1979).

18. Fell, D. & Steele, R. D. Modification of Hepatic Folate Metabolism in rats fed excess retinol. *Life Sci* **38,** 1959–1965 (1986).

19. Schalinske, K. L. & Steele, R. D. Methotrexate alters carbon flow through the hepatic folate-dependent one-carbon pool in rats. *Carcinogenesis* **17,** 1695–1700 (1996).

20. Schalinske, K. L. & Steele, R. D. Quantification of the carbon flow through the folate-dependent one-carbon pool using radiolabeled histidine: effect of altered thyroid and folate status. *Arch. Biochem. Biophys.* **328,** 93–100 (1996).

21. Schirch, D., Villar, E., Maras, B., Barra, D. & Schirch, V. Domain structure and function of 10-formyltetrahydrofolate dehydrogenase. *J Biol Chem* **269,** 24728–24735 (1994).

22. Kutzbach, C. & Stokstad, E. L. R. 10-Formyl tetrahydrofolate: NADP oxidoreductase. *Methods Enzymol.* **18 part B,** 793–798 (1971).

23. Kim, D. W., Huang, T., Schirch, D. & Schirch, V. Properties of tetrahydropteroylpentaglutamate bound to 10-formyltetrahydrofolate dehydrogenase. *Biochemistry* **35,** 15772–83 (1996).

24. Barlowe, C. K. & Appling, D. R. Nitrous oxide exposure reduces hepatic C1-tetrahydrofolate synthase expression in rats. *Biochem.Biophys.Res.Commun.* **157,** 245–249 (1988).

25. Cheek, D. W., Appling, D. R., Cheek, W. D. & Appling, D. R. Purification, Immunoassay, and Tissue Distribution of Rat C1-Tetrahydrofolate Synthase. *Arch. Biochem. Biophys.* **270,** 504–512 (1989).

26. Joyce, B. K., Himes, R. H., Joyce, K. & Himes, H. Formyltetrahydrofolate synthetase. A study of equilibrium reaction rates. *J Biol Chem* **241,** 5716–5724 (1966).

27. Himes, R. H. & Rabinowitz, J. C. Formyltetrahydrofolate synthetase. II. Characteristics of the enzyme and the enzymic reaction. *J Biol Chem* **237,** 2903–2914 (1962).

28. Gardam, M. A., Mejia, N. R. & MacKenzie, R. E. The NADP-dependent trifunctional

methylenetetrahydrofolate dehydrogenase purified from mouse liver is immunologically distinct from the mouse NAD-dependent bifunctional enzyme. *Biochem. Cell Biol.* **66,** 66–70 (1988).

29. Villar, E. *et al.* C1-Tetrahydrofolate synthase from rabbit liver. Structural and kinetic properties of the enzyme and its two domains. *J Biol Chem* **260,** 2245–2252 (1985).

30. Strong, W., Joshi, G., Lura, R., Muthukumaraswamy, N. & Schirch, V. 10-Formyltetrahydrofolate synthetase. Evidence for a conformational change in the enzyme upon binding of tetrahydropteroylpolyglutamates. *J Biol Chem* **262,** 12519–12525 (1987).

31. Curthoys, N. P. & Rabinowitz, J. C. Formyltetrahydrofolate synthetase. Binding of folate substrates and kinetics of the reverse reaction. *J. Biol. Chem.* **247,** 1965–71 (1972).

32. Himes, R. H. & Harmony, J. A. Formyltetrahydrofolate synthetase. *CRC Crit. Rev. Biochem.* **1,** 501–35 (1973).

33. MacKenzie, R. E., Aldridge, M. & Paquin, J. The bifunctional enzyme formiminotransferase-cyclodeaminase is a tetramer of dimers. *J Biol Chem* **255,** 9474–9478 (1980).

34. Paquin, J., Baugh, C. M. & MacKenzie, R. E. Channeling between the active sites of formiminotransferase-cyclodeaminase. Binding and kinetic studies. *J. Biol. Chem.* **260,** 14925–31 (1985).

35. Tabor, H. & Wyngarden, L. The enzymatic formation of formiminotetrahydrofolic acid, 5,10-methenyltetrahydrofolic acid, and 10-formyltetrahydrofolic acid in the metabolism of formiminoglutamic acid. *J Biol Chem* **234,** 1830–1846 (1959).

36. MacKenzie, R. E. Formiminotransferase-cyclodeaminase: a bifunctional enzyme from porcine liver. *Methods Enzym.* **66,** 626–630 (1980).

37. Murley, L. L. & MacKenzie, R. E. The two monofunctional domains of octameric formiminotransferase-cyclodeaminase exist as dimers. *Biochemistry* **34,** 10358–10364 (1995).

38. Findlay, W. A., Zarkadas, C. G. & MacKenzie, R. E. An improved procedure for the purification of formiminotransferase-cyclodeaminase from pig liver. Kinetics of the transferase activity with tetrahydropteroylpolyglutamates. *Biochim. Biophys. Acta* **999,**

52–7 (1989).

39. Daubner, S. C. & Benkovic, S. J. Characterization of mammalian phosphoribosylglycineamide formyltransferase from transformed cells. *Cancer Res.* **45,** 4990–4997 (1985).

40. Caperelli, C. A. Mammalian glycinamide ribonucleotide transformylase: purification and some properties. *Biochemistry* **24,** 1316–1320 (1985).

41. Caperelli, C. A. Mammalian glycinamide ribonucleotide transformylase. Kinetic mechanism and associated de novo purine biosynthetic activities. *J Biol Chem* **264,** 5053–5057 (1989).

42. Wilson, S. D. & Horne, D. W. Effect of nitrous oxide inactivation of vitamin B12 on the levels of folate coenzymes in rat bone marrow, kidney, brain, and liver. *Arch. Biochem. Biophys.* **244,** 248–253 (1986).

43. Eells, J. T., Black, K. A., Makar, A. B., Tephly, T. R. & Tedford, C. E. The Regulation of One-Carbon Oxidation in the Rat by Nitrous Oxide and Methionine. *Arch. Biochem. Biophys.* **219,** 316–326 (1982).

44. Cheng, F. W., Shane, B. & Stokstad, E. L. R. Pentaglutamate Derivatives of Folate as Substrates for Rat Liver Tetrahydropteroylglutamate Methyltransferase and 5,10-Methylenetetrahydrofolate Reductase. *Can. J. Biochem* **53,** 1020–1827 (1975).

45. Yamada, K., Yamada, S., Tobimatsu, T. & Toraya, T. Heterologous high level expression, purification, and enzymological properties of recombinant rat cobalamin-dependent methionine synthase. *J.Biol.Chem.* **274,** 35571–35576 (1999).

46. Chen, Z., Crippen, K., Gulati, S. & Banerjee, R. Purification and kinetic mechanism of a mammalian methionine synthase from pig liver. *J.Biol.Chem.* **269,** 27193–27197 (1994).

47. Matthews, R. G., Ghose, C., Green, J. M., Matthews, K. D. & Dunlap, R. B. Folylpolyglutamates as substrates and inhibitors of folate-dependent enzymes. *Adv. Enzyme Regul.* **26,** 157–171 (1987).

48. Stead, L. M., Au, K. P., Jacobs, R. L., Brosnan, M. E. & Brosnan, J. T. Methylation demand and homocysteine metabolism: effects of dietary provision of creatine and guanidinoacetate. *Am.J.Physiol Endocrinol.Metab* **281,** E1095--E1100 (2001).

49. Jacobs, R. L., Stead, L. M., Brosnan, M. E. & Brosnan, J. T. Hyperglucagonemia in rats results in decreased plasma homocysteine and increased flux through the transsulfuration pathway in liver. *J.Biol.Chem.* **276,** 43740–43747 (2001).

50. Vanoni, M. A., Ballou, D. P. & Matthews, R. G. Methylenetetrahydrofolate reductase. Steady state and rapid reaction studies on the NADPH-methylenetetrahydrofolate, NADPH-menadione, and methyltetrahydrofolate-menadione oxidoreductase activities of the enzyme. *J.Biol.Chem.* **258,** 11510–11514 (1983).

51. Daubner, S. C. & Matthews, R. G. Purification and properties of methylenetetrahydrofolate reductase from pig liver. *J.Biol.Chem.* **257,** 140–145 (1982).

52. Matthews, R. G. & Baugh, C. M. Interactions of pig liver methylenetetrahydrofolate reductase with methylenetetrahydropteroylpolyglutamate substrates and with dihydropteroylpolyglutamate inhibitors. *Biochemistry* **19,** 2040–5 (1980).

53. Vanoni, M. A. & Matthews, R. G. Kinetic isotope effects on the oxidation of reduced nicotinamide adenine dinucleotide phosphate by the flavoprotein methylenetetrahydrofolate reductase. *Biochemistry* **23,** 5272–5279 (1984).

54. Sumner, J., Jencks, D. A., Khani, S. & Matthews, R. G. Photoaffinity labeling of methylenetetrahydrofolate reductase with 8-azido-S-adenosylmethionine. *J.Biol.Chem.* **261,** 7697–7700 (1986).

55. Jencks, D. A. & Mathews, R. G. Allosteric inhibition of methylenetetrahydrofolate reductase by adenosylmethionine. Effects of adenosylmethionine and NADPH on the equilibrium between active and inactive forms of the enzyme and on the kinetics of approach to equilibrium. *J.Biol.Chem.* **262,** 2485–2493 (1987).

56. Matthews, R. G. & Daubner, S. C. Modulation of methylenetetrahydrofolate reductase activity by S-adenosylmethionine and by dihydrofolate and its polyglutamate analogues. *Adv. Enzym. Regul.* **20,** 123–131 (1982).

57. Baggott, J. E. & MacKenzie, R. E. 5,10-methenyltetrahydrofolate cyclohydrolase, rat liver and chemically catalysed formation of 5-formyltetrahydrofolate. *Biochem J* **374,** 773–778 (2003).

58. Bertrand, R., Beauchemin, M., Dayan, A., Ouimet, M. & Jolivet, J. Identification and characterization of human mitochondrial methenyltetrahydrofolate synthetase activity.

Biochim. Biophys. Acta **1266,** 245–249 (1995).

59. Bertrand, R., MacKenzie, R. E. & Jolivet, J. Human liver methenyltetrahydrofolate synthetase: improved purification and increased affinity for folate polyglutamate substrates. *Biochim Biophys Acta* **911,** 154–161 (1987).

60. Hopkins, S. & Schirch, V. 5,10-Methenyltetrahydrofolate synthetase. Purification and properties of the enzyme from rabbit liver. *J Biol Chem* **259,** 5618–5622 (1984).

61. Anguera, M. C., Liu, X. & Stover, P. J. Cloning, expression, and purification of 5,10-methenyltetrahydrofolate synthetase from Mus musculus. *Protein Expr. Purif.* **35,** 276–283 (2004).

62. Huang, T. & Schirch, V. Mechanism for the coupling of ATP hydrolysis to the conversion of 5-formyltetrahydrofolate to 5,10-methenyltetrahydrofolate. *J Biol Chem* **270,** 22296–22300 (1995).

63. Ogawa, H. & Fujioka, M. Purification and characterization of cytosolic and mitochondrial serine hydroxymethyltransferases from rat liver. *J.Biochem.(Tokyo)* **90,** 381–390 (1981).

64. Schirch, L. V *et al.* Serine transhydroxymethylase: evidence for a sequential random mechanism. *Biochemistry* **16,** 410–419 (1977).

65. Masuda, T. *et al.* Affinity purification and characterization of serine hydroxymethyltransferases from rat liver. *J Biochem* **101,** 643–652 (1987).

66. Matthews, R. G., Ross, J., Baugh, C. M., Cook, J. D. & Davis, L. Interactions of pig liver serine hydroxymethyltransferase with methyltetrahydropteroylpolyglutamate inhibitors and with tetrahydropteroylpolyglutamate substrates. *Biochemistry* **21,** 1230–1238 (1982).

67. Ramesh, K. S. & Appaji Rao, N. Purification and physicochemical, kinetic and immunological properties of allosteric serine hydroxymethyltransferase from monkey liver. *Biochem J* **187,** 623–636 (1980).

68. Fu, T. F., Hunt, S., Schirch, V., Safo, M. K. & Chen, B. H. Properties of human and rabbit cytosolic serine hydroxymethyltransferase are changed by single nucleotide polymorphic mutations. *Arch Biochem Biophys* **442,** 92–101 (2005).

69. Schirch, L. & Quashnock, J. Evidence that tetrahydrofolate does not bind to serine

hydroxymethyltransferase with positive homotropic cooperativity. *J Biol Chem* **256,** 6245–6249 (1981).

70. Hashimoto, Y., Shiotani, T., Eble, J. N., Glover, J. L. & Weber, G. Increased thymidylate synthase (EC 2.1.1.45) activity in normal and neoplastic proliferation. *Cancer Biochem.* **10,** 1–10 (1988).

71. Tsukamoto, I., Nakata, R., Miyoshi, M., Taketani, S. & Kojo, S. A new immunoblotting assay for thymidylate synthetase and its application to the regulation of enzyme activity in regenerating rat liver. *Biochim. Biophys. Acta* **964,** 254–259 (1988).

72. Nakata, R., Tsukamoto, I., Miyoshi, M. & Kojo, S. Purification and characterization of thymidylate synthetase from rat regenerating liver. *Biochim Biophys Acta* **924,** 297–302 (1987).

73. Lu, Y. Z., Aiello, P. D. & Matthews, R. G. Studies on the polyglutamate specificity of thymidylate synthase from fetal pig liver. *Biochemistry* **23,** 6870–6876 (1984).

74. Balinska, M., Rhee, M., Whiteley, J. M., Priest, D. G. & Galivan, J. Inhibition of mammalian thymidylate synthase by 10-formyltetrahydropteroylpolyglutamate. *Arch. Biochem. Biophys.* **284,** 219–222 (1991).

75. Stover, P. & Schirch, V. Enzymatic mechanism for the hydrolysis of 5,10-methenyltetrahydropteroylglutamate to 5-formyltetrahydropteroylglutamate by serine hydroxymethyltransferase. *Biochemistry* **31,** 2155–2164 (1992).

Supplementary text S5. Turnover of folate pool in hepatocytes.

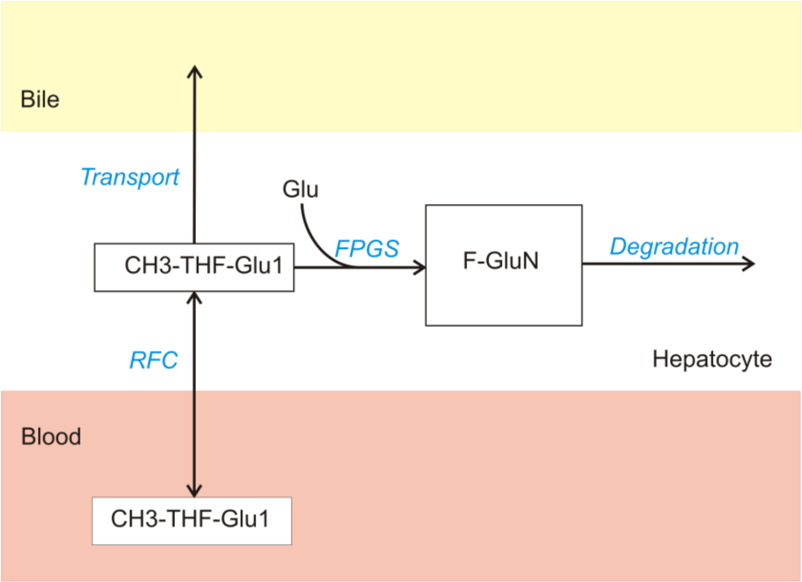

Fig. S5.1. Scheme of folate pool turnover in hepatocytes. Designations: CH3-THF-Glu1 – monoglutamate of methyl-tetrahydrofolate; F-GluN – polyglutamate forms of folates forming intracellular folate pool; Glu – glutamate; RFC – reduced folate carrier transporting monoglutamate forms of folates, FPGS – folylpolyglutamate synthase.

The main circulating form of folates is monoglutamate of methyl-tetrahydrofolate (CH3-THF-Glu1). Under physiological conditions it enters hepatocytes primarily via the anion exchange transporter - reduced folate carrier (RFC) [1] (Fig. S5.1). Once in the hepatocyte, CH3-THF-Glu1 can be transported from the hepatocyte to the bile ducts, or it can be converted to polyglutamate forms by the enzyme folylpolyglutamate synthase (FPGS) [2]. Intracellular folate pool consists of the polyglutamate forms containing 5-6 glutamate residues per folate molecule. The polyglutamate forms do not pass through cell membrane and are much more efficient substrates for enzymes of folate metabolism than free folates or monoglutamate forms [2]. There are no known specific mechanisms of intracellular folates degradation and most likely intracellular folates degrade via slow non-enzymatic oxidation. While there is a lysosomal enzyme gamma-glutamyl hydrolase [3] which cleaves glutamate residues from folates, it probably helps to degrade already damaged oxidized folates. Folates that get into the bile are transported to the intestine where they are re-absorbed to blood and again enter the liver through the portal vein. Thus, folates are involved in entero-hepatic circulation [4,5].

The kinetics of folate pool can be described by the following equation:

$$\frac{dF}{dt} = V^{FPGS} - V^{degr} \tag{S5.1}$$

where F is folate pool (sum of concentrations of polyglutamate forms of folates), V^{FPGS} is the rate of folylpolyglutamate synthase, V^{degr} is the rate of non-enzymatic folate pool degradation. The rate for non-enzymatic folate degradation can be described as follows:

$$V^{degr} = k^{degr} \cdot F \tag{S5.2}$$

where k^{degr} is reaction rate constant for the non-enzymatic folate pool degradation. The estimation for the rate constant k^{degr} of folate pool degradation at physiological conditions can be obtained from experiments with prolonged feeding of rats with folate-free diet. [6,7]. The initial drop in folate concentration in rat liver gives a rate of decrease in folate pool (*Vdegr*) equal to 0.06 µmol/h kg liver. It gives us a value for the rate constant $k^{degr} = V^{degr}/F = 0.06/20 = 3*10^{-3}$ h^{-1}. Under steady-state conditions the rate of folate pool degradation is equal to the rate of its accumulation:

$$V^{FPGS} = k^{degr} \cdot F \tag{S5.3}$$

That gives us an estimation for the characteristic time of folate pool turnover that is equal to $1/k^{degr}$ = 333 h. This time is several orders of magnitude bigger than characteristic times of folate metabolites and folate dependent processes (Table 2, 5). Thus for all metabolic processes associated with folate metabolism in our model we can consider folate pool as a constant model parameter.

References

1. Zhao, R., Matherly, L. H. & Goldman, I. D. Membrane transporters and folate homeostasis: intestinal absorption and transport into systemic compartments and tissues. *Expert Rev. Mol. Med.* **11,** e4 (2009).

2. Shane, B. Folylpolyglutamate synthesis and role in the regulation of one-carbon metabolism. *Vitam. Horm.* **45,** 263–335 (1989).

3. Schneider, E. & Ryan, T. J. Gamma-glutamyl hydrolase and drug resistance. *Clin. Chim.*

Acta **374,** 25–32 (2006).

4. Roberts, M. S., Magnusson, B. M., Burczynski, F. J. & Weiss, M. Enterohepatic Circulation. Physiological , Pharmacokinetic and Clinical Implications. **41,** 751–790 (2002).

5. Steinberg, S. E., Campbell, C. L. & Hillman, R. S. Kinetics of the normal folate enterohepatic cycle. *J Clin Invest* **64,** 83–88 (1979).

6. McGuffin, R., Goff, P. & Hillman, R. S. The effect of diet and alcohol on the development of folate deficiency in the rat. *Br. J. Haematol.* **31,** 185–92 (1975).

7. Chanarin, I., Smith, G. N. & Wincour, V. Development of folate deficiency in the rat. *Br. J. Haematol.* **16,** 193–5 (1969).
